# Finite mixtures of matrix variate Poisson-log normal distributions for three-way count data

**DOI:** 10.1093/bioinformatics/btad167

**Published:** 2023-04-05

**Authors:** Anjali Silva, Xiaoke Qin, Steven J Rothstein, Paul D McNicholas, Sanjeena Subedi

**Affiliations:** Department of Mathematics and Statistics, University of Guelph, Guelph, ON N1G 2W1, Canada; Department of Molecular and Cellular Biology, University of Guelph, Guelph, ON N1G 2W1, Canada; School of Mathematics and Statistics, Carleton University, Ottawa, ON K1S 5B6, Canada; Department of Molecular and Cellular Biology, University of Guelph, Guelph, ON N1G 2W1, Canada; Department of Mathematics and Statistics, McMaster University, Hamilton, ON L8S 4L8, Canada; School of Mathematics and Statistics, Carleton University, Ottawa, ON K1S 5B6, Canada

## Abstract

**Motivation:**

Three-way data structures, characterized by three entities, the units, the variables and the occasions, are frequent in biological studies. In RNA sequencing, three-way data structures are obtained when high-throughput transcriptome sequencing data are collected for *n* genes across *p* conditions at *r* occasions. Matrix variate distributions offer a natural way to model three-way data and mixtures of matrix variate distributions can be used to cluster three-way data. Clustering of gene expression data is carried out as means of discovering gene co-expression networks.

**Results:**

In this work, a mixture of matrix variate Poisson-log normal distributions is proposed for clustering read counts from RNA sequencing. By considering the matrix variate structure, full information on the conditions and occasions of the RNA sequencing dataset is simultaneously considered, and the number of covariance parameters to be estimated is reduced. We propose three different frameworks for parameter estimation: a Markov chain Monte Carlo-based approach, a variational Gaussian approximation-based approach, and a hybrid approach. Various information criteria are used for model selection. The models are applied to both real and simulated data, and we demonstrate that the proposed approaches can recover the underlying cluster structure in both cases. In simulation studies where the true model parameters are known, our proposed approach shows good parameter recovery.

**Availability and implementation:**

The GitHub R package for this work is available at https://github.com/anjalisilva/mixMVPLN and is released under the open source MIT license.

## 1 Introduction

Finite mixture models are popular for clustering applications and are widely used on two-way data (McLachlan and Peel 2000; McNicholas 2016). Three-way data are becoming increasingly commonplace in several fields, including bioinformatics. Three-way data structures are characterized by three entities or modes: the units (rows), the variables (columns), and the occasions (layers). For two-way data, each observation is represented as a vector whereas, for three-way data, each observation can be regarded as a matrix. A random matrix $T_{n}$ is said to contain k∈{1,…,p} responses over i∈{1,…,r} occasions and n=1,…,N such units are considered. This provides *N* independent and identically distributed random matrices $T_{1},T_{2},\ldots ,T_{N}$.

Matrix variate distributions offer a natural approach for modeling three-way data. Extensions of matrix variate distributions in the context of mixture models have given rise to mixtures of matrix variate distributions, which have been used to cluster three-way data (Viroli 2011; Anderlucci and Viroli 2015; Dogru et al. 2016; Gallaugher and McNicholas 2018). Here, the interest lies in clustering the *N* observed matrices into *G* clusters, while utilizing all information from the other two modes (Viroli 2011). It is assumed that matrices $T_{1},T_{2},\ldots ,T_{N}$ are conditionally independent and identically distributed observations coming from a mixture model with *G* possible groups in proportions $\pi_{1},\ldots ,\pi_{G}$ (Viroli 2011). The density of the *G*-component mixture is $f(T_{n}|\pi_{1},\ldots ,\pi_{G},\vartheta_{1},\ldots ,\vartheta_{G})=\sum_{g=1}^{G}\pi_{g}f^{\left(r\times p\right)}(T_{n}|\vartheta_{g}).$ Here parameters of the distribution function $f^{\left(r\times p\right)}(\cdot )$ are represented by $\vartheta_{g}$ and $\pi_{g}>0$, such that $\sum_{g=1}^{G}\pi_{g}=1$, is the mixing proportion of the *g*th component.

Three-way datasets are common in biological studies, including RNA sequencing (RNA-seq), where gene expression count data are collected for *N* genes across *p* conditions at *r* occasions. However, efficiently analyzing these complex data remains an ongoing challenge. While some early work utilized the Poisson distribution to model such count data (Marioni et al. 2008; Bullard et al. 2010), this was not ideal because of its restrictive mean–variance relationship, and so the negative binomial distribution emerged as the univariate distribution of choice (Love et al. 2014; Dong et al. 2016). However, the multivariate extensions of Poisson (Campbell 1934) and negative binomial distributions (Doss 1979) are seldom used in practice due to their computational complexity (Brijs et al. 2004). Silva et al. (2019) proposed a mixture model-based clustering methodology for overdispersed, multivariate count data based on the multivariate Poisson-log normal (MPLN) distribution for two-way RNA-seq data. For genes n∈{1,…,N} and samples c∈{1,…,rp}, the MPLN distribution is given by


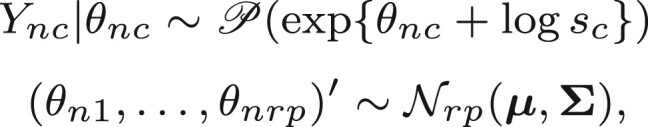


where 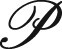 denotes the Poisson distribution and the $N_{rp}$ is a *rp*-dimensional normal distribution. To account for the differences in library sizes across each sample *c* of an RNA-seq study, a fixed, known constant *s_c_*, representing the normalized library sizes, is added to the mean of the Poisson distribution. In this work, mixtures of MPLN distributions and matrix variate normal distributions are extended to give rise to mixtures of matrix variate Poisson-log normal (MVPLN) distributions for clustering three-way count data. Details of parameter estimation are provided, and both real and simulated data illustrations are used to demonstrate the clustering ability.

## 2 Materials and methods

### 2.1 Matrix variate Poisson-log normal distribution

**Figure 1.**
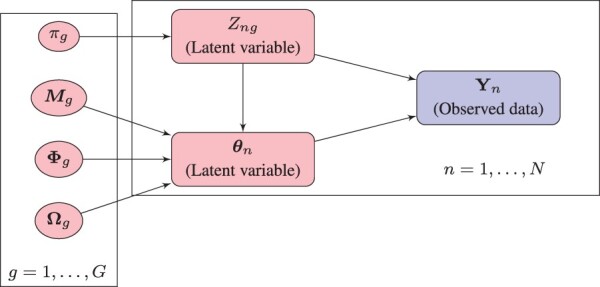
Graphical representation of the MVPLN mixture model.

Mathematical properties of the matrix variate normal distribution can be found in Gupta and Nagar (2000). By considering a matrix variate structure, the number of free covariance parameters to be estimated is reduced from (1/2)rp(rp+1) to (1/2)[r(r+1)+p(p+1)]. The matrix variate normal distribution can be extended to give rise to MVPLN distribution using a hierarchical structure. Consider *N* independent and identically distributed random matrices $Y_{n},\;n=1,\ldots ,N$, each of dimension *r *×* p*. In the MVPLN framework, $Y_{\mathrm{nik}}|\theta_{\mathrm{nik}}$ follows a Poisson distribution with mean $\text{exp}(\theta_{\mathrm{nik}})$, and ${\theta_{n}}^{}$ follows a *r *×* p* matrix variate normal distribution $N_{r\times p}(M,\Phi ,\Omega )$, where **M** is a *r *×* p* matrix of means, Φ is a *r *×* r* covariance matrix containing the variances and covariances between *r* occasions, and Ω is a *p *×* p* covariance matrix containing the variance and covariances of the *p* variables. Figure 1 provides a graphical representation of a mixture of MVPLN distributions.

The vectorization of ${Y_{n}}^{⊤}$, denoted $\text{vec}({Y_{n}}^{⊤})$ , is *rp*-dimensional. Given all $\text{vec}({Y_{n}}^{⊤})$, i.e. for n=1,…,N, the library sizes vec(s) can be calculated. The vec(s) and $\text{vec}({\theta_{n}}^{⊤})$ are *rp*-dimensional. The covariance matrix of $\text{vec}({Y_{n}}^{⊤})$ is Σ=Φ⊗Ω, where ⊗ denotes the Kronecker product. Note that Σ has dimension *rp* × *rp*, and the probability mass function of the MVPLN distribution is where ϑ=(M,Φ,Ω), f(·) is the probability mass function of Poisson distribution, $\mathrm{vec}{\left({Y_{n}}^{⊤}\right)}_{c}$ represent the $c^{th}$ element of $\mathrm{vec}({Y_{n}}^{⊤}),$ and $g^{\left(r\times p\right)}(\cdot )$ is the probability density function of matrix variate normal distribution.


$$\begin{array}{l} f(Y_{n},s|\vartheta )=\\ \int_{\mathbb{R}}\left\{\prod_{c=1}^{rp}f(\text{vec}({Y_{n}}^{⊤}{)}_{c}|\text{vec}({\theta_{n}}^{⊤}{)}_{c},\text{vec}(s{)}_{c})\right\}g^{\left(r\times p\right)}(\theta_{n}|\vartheta )\;d\text{vec}({\theta_{n}}^{⊤}), \end{array}$$

The unconditional mean and covariance of the MPLN distribution can be calculated using the properties of the log-normal distribution and of the conditional expectation (Aitchison and Ho 1989; Tunaru 2002). For the MVPLN distribution, the unconditional mean and covariance are respectively. The MVPLN distribution can account for both the correlations between variables and the correlations between occasions, as two different covariance matrices are used for the two modes. This makes the model ideal for modeling RNA-seq data when expression measurements for different conditions at different occasions, e.g. time-points or replicates, are available.


$$\begin{array}{l} E(Y_{ik})=E[E(Y_{ik}|\theta_{ik})]=\text{exp}\;\left\{\mu_{ik}+\frac{1}{2}(\Phi_{ii}\Omega_{kk})\right\}=M_{ik},\\ V\text{ar}(Y_{ik})=E[V\text{ar}(Y_{ik}|\theta_{ik})]+V\text{ar}[E(Y_{ik}|\theta_{ik})]\\ =M_{ik}+M_{ik}^{2}\left(\text{exp}\;\{\Phi_{ii}\Omega_{kk}\}-1\right), \end{array}$$

### 2.2 Finite mixtures of MVPLN distributions

**Figure 2.**
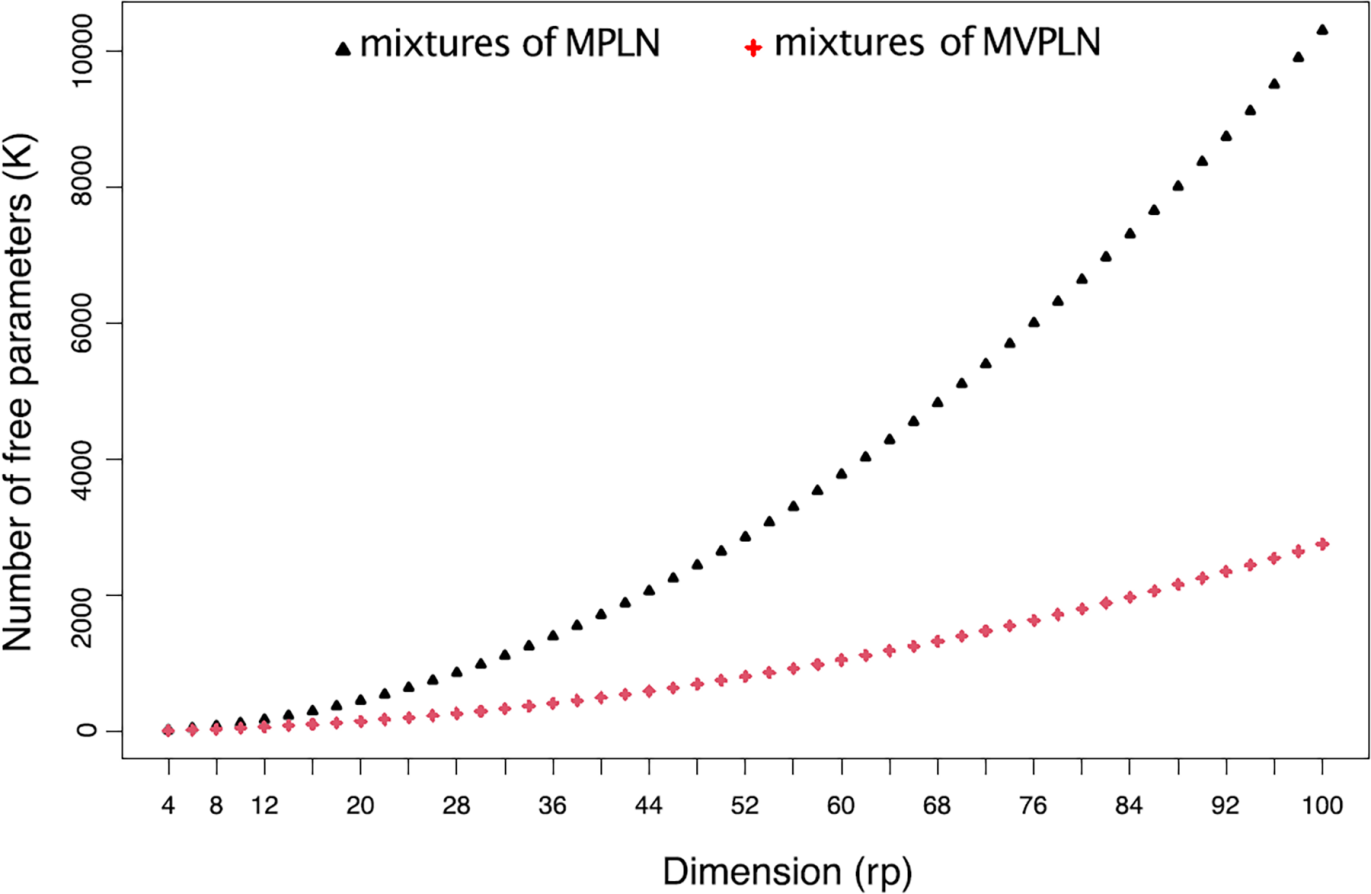
Scatter plot illustrating how the number of free parameters *K* grows with data dimensionality *rp* for the mixtures of MPLN model and for the mixtures of MVPLN model. Here G=2,r=2, and *rp *=* *4 up to 100.

**Figure 3.**
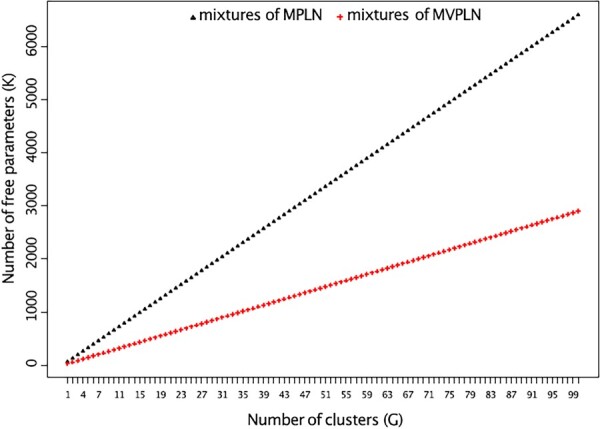
Scatter plot illustrating how the number of free parameters *K* grows with the number of clusters *G* for the mixtures of MPLN model and for the mixtures of MVPLN model. Here G=1:100,r=2,p=5.

In the mixture model context, a random matrix $Y_{n}$ is assumed to come from a population with *G* subgroups each distributed according to an MVPLN distribution. Then *N* such matrices $Y_{1},Y_{2},\ldots ,Y_{N}$ are observed, each of which belongs to one of g∈{1,…,G} different sub-populations with mixing proportions $\pi_{1},\ldots ,\pi_{G}$. Then the probability density function of a *G*-component mixture of MVPLN distributions can be written as where $\Theta =(\pi_{1},\ldots ,\pi_{G},M_{1},\ldots ,M_{G},\Phi_{1},\ldots ,\Phi_{G},\Omega_{1},\ldots ,\Omega_{G})$, the $f_{g}(\cdot )$ is the probability mass function of a Poisson distribution and the $g_{g}^{\left(r\times p\right)}(\cdot )$ is the probability density function of matrix variate normal distribution. The cluster membership of all units is assumed to be unknown and *z_ng_* is used to cluster membership, where *z_ng_* = 1 if $Y_{n}$ is in component *g* and *z_ng_* = 0 otherwise. The complete data consist of the observed and missing data, i.e. ($Y_{1},\ldots ,Y_{N},z_{1},\ldots ,z_{N},\theta_{1},\ldots ,\theta_{N}$). The complete-data likelihood is and the complete-data log-likelihood is where $n_{g}=\sum_{n=1}^{N}z_{ng}$ and $\text{log (vec}{\left({Y_{n}}^{⊤}\right)}_{c}\text{!)}$ is the log of the factorial of the $c^{th}$ element of $\mathrm{vec}({Y_{n}}^{⊤}).$Compared to the mixtures of MPLN distribution, the number of free parameters to be estimated is reduced by considering a matrix variate structure (see Figs 2 and 3). For the mixtures of MPLN model, the number of free parameters is $K=(G-1)+(\mathrm{Grp})+\frac{1}{2}Grp[rp+1]$, whereas for mixtures of MVPLN model it is $K=(G-1)+(\mathrm{Grp})+\frac{1}{2}G[r(r+1)+p(p+1)]$.


$$\begin{array}{l} f(Y;\Theta )=\sum_{g=1}^{G}\pi_{g}f_{Y}(Y|M_{g},\Phi_{g},\Omega_{g})\\ =\sum_{g=1}^{G}\pi_{g}\int_{\mathbb{R}}\left\{\prod_{c=1}^{rp}f_{g}(\text{vec}({Y_{n}}^{⊤}|\text{vec}({\theta_{ng}}^{⊤}{)}_{c},\text{vec}(s{)}_{c})\right\}\\ \times g_{g}^{\left(r\times p\right)}(\theta_{ng}|M_{g},\Phi_{g},\Omega_{g})\;d\text{vec}({\theta_{ng}}^{⊤}) \end{array}$$

$$\begin{array}{l} L_{c}(\Theta )=\prod_{n=1}^{N}\prod_{g=1}^{G}[\pi_{g}\left\{\prod_{c=1}^{rp}f_{g}(\text{vec}({Y_{n}}^{⊤}|\text{vec}({\theta_{ng}}^{⊤}{)}_{c},\text{vec}(s{)}_{c})\right\}\\ \times \;g_{g}^{\left(r\times p\right)}(\theta_{ng}|M_{g},\Phi_{g},\Omega_{g}){]}^{z_{ng}}, \end{array}$$

$$\begin{array}{l} l_{c}(\Theta )=\sum_{g=1}^{G}n_{g}\;\text{log}\;\pi_{g}\\ -\;\sum_{n=1}^{N}\sum_{g=1}^{G}\sum_{c=1}^{rp}z_{ng}\;\text{exp}\;\{\text{vec}{\left({\theta_{ng}}^{⊤}\right)}_{c}+\text{log}\;\text{vec}{\left(s\right)}_{c}\}\\ +\;\sum_{n=1}^{N}\sum_{g=1}^{G}z_{ng}\left[\text{vec}({\theta_{ng}}^{⊤})+\text{log}\;\text{vec}(s)\right]\text{vec}{\left({Y_{n}}^{⊤}\right)}^{⊤}\\ -\;\sum_{n=1}^{N}\left(\sum_{g=1}^{G}z_{ng}\right)\sum_{c=1}^{rp}\;\text{log}\;(\text{vec}{\left({Y_{n}}^{⊤}\right)}_{c}\text{!})-\frac{\mathrm{nrp}}{2}\text{log}(2\pi )\\ -\;\frac{p}{2}\sum_{g=1}^{G}n_{g}\;\text{log}\;|\Phi_{g}|-\frac{r}{2}\sum_{g=1}^{G}n_{g}\;\text{log}\;|\Omega_{g}|\\ -\;\frac{1}{2}\sum_{n=1}^{N}\sum_{g=1}^{G}z_{ng}\text{tr}\left[\Phi_{g}^{-1}(\theta_{ng}-M_{g})\Omega_{g}^{-1}{\left(\theta_{ng}-M_{g}\right)}^{⊤}\right], \end{array}$$

### 2.3 Parameter estimation

Three different frameworks for parameter estimation for the mixtures of MVPLN models are proposed: one based on Markov chain Monte Carlo (MCMC) methods, one based on variational Gaussian approximation (VGA) as well as a hybrid approach. MCMC-based approaches are computationally intensive; hence, we also provide a computationally efficient variational approximation framework for parameter estimation. Finally, a hybrid approach combines the variational approximation-based approach and MCMC-based approach.

#### 2.3.1 MCMC-based approach

In the MCMC-based approach, the MCMC expectation–maximization (MCMC–EM) algorithm is used to estimate the model parameters (see Silva et al. 2019, for details). Using an MCMC–EM algorithm, the expected value of the $\theta_{ng}$ and the *Z_ng_* conditional on the parameter updates from the *t*th iteration, respectively, are updated in the expectation (E-) step as follows: where and $\theta_{ng}^{\left(f\right)}$ is a random sample simulated via the RStan package for iterations f=1,…,B. In the E-step, the expectation is taken conditional on the current parameter estimates; hence, the use of (*t*) on parameters in (1). As the values from initial iterations are discarded from further analysis to minimize bias, the number of iterations used for parameter estimation is *W*, where *W *<* B*. The conditional expected-value of the complete-data log-likelihood is where *C* is a constant with respect to $M_{g},\;\Phi_{g}$ and $\Omega_{g}$, and $n_{g}^{\left(t\right)}=\sum_{n=1}^{N}z_{ng}^{\left(t\right)}$. During the M-step, the updates for the parameters are obtained as follows:


(1) $$\begin{array}{l} E(\theta_{ng}|Y_{n})≃\frac{1}{W}\sum_{f=1}^{W}\theta_{ng}^{\left(f\right)}≃\theta_{ng}^{\left(t\right)},\\ E(Z_{ng}|Y_{n},\theta_{ng},s)=\frac{q_{ng}}{\sum_{h=1}^{G}q_{nh}}:=z_{ng}^{\left(t\right)}, \end{array}$$

$$\begin{array}{l} q_{ng}=\pi_{g}^{\left(t\right)}\left\{\prod_{c=1}^{rp}f_{g}\left(\text{vec}{\left({Y_{n}}^{⊤}\right)}_{c}|\text{vec}{\left({\theta_{ng}}^{⊤}\right)}_{c},\text{vec}{\left(s\right)}_{c}\right)\right\}\\ \;\;\;\;\;\;\times g_{g}^{\left(r\times p\right)}(\theta_{ng}^{\left(t\right)}|M_{g}^{\left(t\right)},\Phi_{g}^{\left(t\right)},\Omega_{g}^{\left(t\right)}) \end{array}$$

$$\begin{array}{l} Q(\Theta )≃E\{l_{c}(\Theta )\}≃C-\frac{p}{2}\sum_{g=1}^{G}n_{g}\;\text{log}\;|\Phi_{g}|-\frac{r}{2}\sum_{g=1}^{G}n_{g}\;\text{log}\;|\Omega_{g}|\\ -\frac{1}{2}\sum_{n=1}^{N}\sum_{g=1}^{G}z_{ng}^{\left(t\right)}E[\text{tr}(\Phi_{g}^{-1}(\theta_{ng}-M_{g})\Omega_{g}^{-1}{\left(\theta_{ng}-M_{g}\right)}^{⊤})|\theta_{ng}^{\left(t\right)},z_{ng}=1], \end{array}$$

$$\begin{array}{l} \pi_{g}^{\left(t+1\right)}=\frac{n_{g}^{\left(t\right)}}{N},\;M_{g}^{\left(t+1\right)}=\frac{\sum_{n=1}^{N}z_{ng}^{\left(t\right)}E(\theta_{ng})}{n_{g}},\\ \Phi_{g}^{\left(t+1\right)}=\frac{\sum_{n=1}^{N}z_{ng}^{\left(t\right)}E((\theta_{ng}-M_{g}^{\left(t+1\right)}){\left(\Omega_{g}^{\left(t\right)}\right)}^{-1}{\left(\theta_{ng}-M_{g}^{\left(t+1\right)}\right)}^{⊤})}{pn_{g}},\\ \Omega_{g}^{\left(t+1\right)}=\frac{\sum_{n=1}^{N}z_{ng}^{\left(t\right)}E({\left(\theta_{ng}-M_{g}^{\left(t+1\right)}\right)}^{⊤}{\left(\Phi_{g}^{\left(t+1\right)}\right)}^{-1}(\theta_{ng}-M_{g}^{\left(t+1\right)}))}{rn_{g}}. \end{array}$$

#### 2.3.2 VGA-based approach

Variational approximations (Wainwright and Jordan 2007) are approximate inference techniques in which a computationally convenient approximating density is used in place of a more complex but “true” posterior density. The approximating density is obtained by minimizing the Kullback–Leibler (KL) divergence between the true and the approximating densities. Suppose we have an approximating density q(θ), then the marginal log of the probability mass function can be written where $D_{\text{KL}}(q||f)=\int q(\theta )\;\text{log}\;\frac{q(\theta )}{f(\theta |Y)}d\theta$ is the KL divergence between f(θ|Y) and approximating distribution q(θ), and F(Y,q)=∫[ log f(Y,θ)−log q(θ)]q(θ)dθ is our evidence lower bound (ELBO). Thus, to minimize the KL divergence, we maximize our ELBO. For VGA, q(θ) is assumed to be a Gaussian distribution.


$$\text{log}\;f_{Y}(Y)=F(q,Y)+D_{\text{KL}}(q||f_{Y}),$$

The complete-data log-likelihood for the mixtures of MVPLN distributions can be written


$$\begin{array}{l} l_{c}(\Theta )=\sum_{g=1}^{G}\sum_{n=1}^{N}z_{ng}\;\text{log}\;\pi_{g}+\sum_{g=1}^{G}\sum_{n=1}^{N}z_{ng}\;\text{log}\;f(Y_{n}|M_{g},\Phi_{g},\Omega_{g})\\ =\sum_{g=1}^{G}\sum_{n=1}^{N}z_{ng}\;\text{log}\;\pi_{g}+\sum_{g=1}^{G}\sum_{n=1}^{N}z_{ng}\left[F(Y_{n},q_{ng})+D_{\text{KL}}(q_{ng}||f_{ng})\right], \end{array}$$

where $D_{\text{KL}}(q_{ng}||f_{ng})=\int q(\theta_{ng})\;\text{log}\;\frac{q(\theta_{ng})}{f(\theta_{n}|Y_{n},Z_{ng}=1)}d\theta_{ng}$ is the KL divergence between $f(\theta_{n}|Y_{n},Z_{ng}=1)$ and approximating distribution $q(\theta_{ng})$. Assuming $q(\theta_{ng})=N_{r\times p}(\xi_{ng},\Delta_{ng},\kappa_{ng})$, the ELBO for each observation $y_{n}$ becomes


$$\begin{array}{l} F(q_{ng},Y_{n})=-[\sum_{i=1}^{r}\sum_{j=1}^{p}\exp\{{\left(\xi_{ng}\right)}_{ij}+\frac{1}{2}{\left(\Delta_{ng}\right)}_{ii}{\left(\Omega_{ng}\right)}_{jj}+\log s_{ij}\}]\\ +\;{\left[\text{vec}\left(\xi_{ng}^{⊤}\right)+\log\;\text{vec}(s)\right]}^{⊤}\text{vec}(Y_{n}^{⊤})\\ -\;\left[\sum_{c=1}^{rp}\log(\text{vec}{\left(Y_{n}^{⊤}\right)}_{c}!)\right]-\frac{p}{2}\log|\Phi_{g}|-\frac{r}{2}\log|\Omega_{g}|\\ -\;\frac{1}{2}{\left(\text{vec}(\xi_{ng}^{⊤})-\text{vec}(M_{g}^{⊤})\right)}^{⊤}\Phi_{g}^{-1}⊗\Omega_{g}^{-1}\left(\text{vec}(\xi_{ng}^{⊤})-\text{vec}(M_{g}^{⊤})\right)\\ +\;\text{tr}\left\{\Phi_{g}^{-1}\Delta_{ng}\right\}\text{tr}\left\{\Omega_{g}^{-1}\kappa_{ng}\right\}+\frac{p}{2}\log|\Delta_{ng}|+\frac{r}{2}\log|\kappa_{ng}|+\frac{rp}{2}. \end{array}$$

The variational parameters that maximize the ELBO will minimize the KL divergence between the true posterior and the approximating density. Thus, parameter estimation can be done in an iterative EM-type approach such that the following steps are iterated. At the (*t* + 1)th step:

1. Conditional on the variational parameters $\xi_{ng},\;\Delta_{ng}$, and $\kappa_{ng}$ and on $M_{g},\;\Phi_{g}$, and $\Omega_{g}$, the $E(Z_{ng})$ is computed. Given *π_g_*, $M_{g},\;\Phi_{g}$, and $\Omega_{g}$,
  $$E(Z_{ng}|Y_{n})=\frac{\pi_{g}f(Y_{n}|M_{g},\Phi_{g},\Omega_{g})}{\sum_{h=1}^{G}\pi_{h}f(Y_{n}|M_{h},\Phi_{h},\Omega_{h})}.$$
  Note that this involves the marginal distribution of **Y** which is difficult to compute. Hence, we use an approximation of $E(Z_{ng})$, where we replace the marginal density of the exponent of ELBO such that
  $${\hat{z}}_{ng}^{\left(t+1\right)}\overset{\text{def}}{=}\frac{\pi_{g}\;\text{exp}\;\left[F\left(q_{ng},Y_{n}\right)\right]}{\sum_{h=1}^{G}\pi_{h}\;\text{exp}\;\left[F\left(q_{nh},Y_{n}\right)\right]}.$$
  This approximation is computationally convenient and a similar framework has been previously utilized (Gollini and Murphy 2014; Tang et al. 2015). This approximation works well in simulation studies and real data analysis.
2. Given ${\hat{z}}_{ng}^{\left(t+1\right)}$, variational parameters $\xi_{ng},\;\Delta_{ng}$, and $\kappa_{ng}$ are updated conditional on $M_{g}^{\left(t\right)},\;\Phi_{g}^{\left(t\right)}$, and $\Omega_{g}^{\left(t\right)}$.
  - A fixed-point method is used for updating $\Delta_{n}$:
    $$\begin{array}{l} \Delta_{ng}^{\left(t+1\right)}=p[I_{r\times r}⊙\{\text{diag}{\left(\kappa_{ng}^{\left(t\right)}\right)}^{⊤}[exp\{\xi_{ng}^{\left(t\right)}+\text{log}\;s\\ {{+\frac{1}{2}\text{diag}(\Delta_{ng}^{\left(t\right)})\text{diag}{\left(\kappa_{ng}^{\left(t\right)}\right)}^{⊤}\}]}^{⊤}\}+\Phi_{g}^{-1(t)}\text{tr}\left\{\Omega_{g}^{-1(t)}\kappa_{ng}^{\left(t\right)}\}\right]}^{-1}, \end{array}$$
    where the vector function $exp\left[a\right]=(e^{a_{1}},\ldots ,e^{a_{r}})\prime$ is a vector of the exponential of each element of the *r*-dimensional vector **a**, $\text{diag}(\kappa )=(\kappa_{11}\ldots ,\kappa_{pp})$ puts the diagonal elements of the *p *×* p* matrix κ into a *p*-dimensional vector, and ⊙ is the Hadmard product.
  - A fixed-point method is used for updating $\kappa_{ng}$:
    $$\begin{array}{l} \kappa_{ng}^{\left(t+1\right)}=r[I_{p\times p}⊙\{\text{diag}{\left(\Delta_{ng}^{\left(t+1\right)}\right)}^{⊤}[exp\{\xi_{ng}^{\left(t\right)}+\text{log}\;s\\ {+\frac{1}{2}{\left(\text{diag}(\kappa_{ng}^{\left(t\right)})\text{diag}{\left(\Delta_{ng}^{\left(t+1\right)}\right)}^{⊤}\right)}^{⊤}\}]\}+\Omega_{g}^{-1(t)}\text{tr}\left\{\Phi_{g}^{-1(t)}\Delta_{ng}^{\left(t+1\right)}\right\}]}^{-1}, \end{array}$$
    where the vector function $exp\left[a\right]=(e^{a_{1}},\ldots ,e^{a_{p}})\prime$ is a vector of exponential each element of the *p*-dimensional vector **a**, $\text{diag}(\Delta )=(\Delta_{11}\ldots ,\Delta_{rr})$ puts the diagonal elements of the *r *×* r* matrix Δ into a *r*-dimensional vector, and ⊙ is the Hadmard product.
  - Newton’s method is used to update $\xi_{ng}$:
    $$\begin{array}{l} \text{vec}(\xi_{ng}^{⊤(t+1)})=\text{vec}(\xi_{ng}^{⊤(t)})-\Psi_{ng}^{-1(t+1)}\{\text{vec}(Y_{n}^{⊤})-exp[\log\;\text{vec}(s^{⊤})\\ \;\;\;\;\;\;\;\;\;\;\;\;\;\;\;\;\;\;\;\;\;+\text{vec}(\xi_{ng}^{⊤(t)})+\frac{1}{2}\text{diag}\left(\Psi_{ng}^{-1(t+1)})\right]\\ \;\;\;\;\;\;\;\;\;\;\;\;\;\;\;\;\;\;\;\;\;-\Psi_{ng}^{-1(t+1)}\left(\text{vec}(\xi_{ng}^{⊤(t)})-\text{vec}(M_{g}^{⊤(t)})\right)-\text{vec}(Y_{n}^{⊤})\}, \end{array}$$
    where $\Psi_{ng}^{\left(t+1\right)}=\Delta_{ng}^{\left(t+1\right)}⊗\kappa_{ng}^{\left(t+1\right)}$.
3. Given ${\hat{z}}_{ng}^{\left(t+1\right)}$ and the variational parameters $\xi_{ng}^{\left(t+1\right)},\;\Delta_{ng}^{\left(t+1\right)}$, and $\kappa_{ng}^{\left(t+1\right)}$, the parameters *π_g_*, $M_{g},\;\Phi_{g}$, and $\Omega_{g}$ can be solved for as
  $$\begin{array}{l} \pi_{g}^{\left(t+1\right)}=\frac{n_{g}^{\left(t+1\right)}}{N}\;\text{where}\;n_{g}^{\left(t+1\right)}=\sum_{n=1}^{N}{\hat{z}}_{ng}^{\left(t+1\right)},\\ M_{g}^{\left(t+1\right)}=\frac{\sum_{n=1}^{N}{\hat{z}}_{ng}^{\left(t+1\right)}\xi_{ng}^{\left(t+1\right)}}{n_{g}^{\left(t+1\right)}},\\ \Phi_{g}^{\left(t+1\right)}=\frac{\sum_{n=1}^{N}{\hat{z}}_{ng}^{\left(t+1\right)}(\xi_{ng}^{\left(t+1\right)}-M_{g}^{\left(t+1\right)})\Omega_{g}^{-1(t)}{\left(\xi_{ng}^{\left(t+1\right)}-M_{g}^{\left(t+1\right)}\right)}^{⊤}}{pn_{g}^{\left(t+1\right)}}\\ +\frac{\sum_{n=1}^{N}{\hat{z}}_{ng}^{\left(t+1\right)}\Delta_{ng}^{\left(t+1\right)}\text{tr}\left\{\Omega^{-1(t)}\kappa_{ng}^{\left(t+1\right)}\right\}}{pn_{g}^{\left(t+1\right)}},\\ \Omega_{g}^{\left(t+1\right)}=\frac{\sum_{n=1}^{N}{\hat{z}}_{ng}^{\left(t+1\right)}\kappa_{ng}^{\left(t+1\right)}\text{tr}\left\{\Phi_{g}^{-1(t+1)}\Delta_{ng}^{\left(t+1\right)}\right\}}{rn_{g}^{\left(t+1\right)}}\\ +\frac{\sum_{n=1}^{N}{\hat{z}}_{ng}^{\left(t+1\right)}{\left(\xi_{ng}^{\left(t+1\right)}-M_{g}^{\left(t+1\right)}\right)}^{⊤}\Phi_{g}^{-1(t+1)}(\xi_{ng}^{\left(t+1\right)}-M_{g}^{\left(t+1\right)})}{rn_{g}^{\left(t+1\right)}}. \end{array}$$

#### 2.3.3 Hybrid approach

While the MCMC-based approach can generate exact results, fitting such models can be computationally intensive because we need to evaluate the expected complete-data log-likelihood with respect to the posterior distribution of the latent variables at every iteration of the EM algorithm. For example, for datasets with *N *=* *1000, *rp *=* *6, and *G *=* *2 in Simulation setting 2, the MCMC-based approach took on an average of ∼41 h. On the other hand, the VGA approach is computationally efficient—on the same set of datasets from Simulation 2 with N=1000,rp=6, and *G *=* *2 fitting such a model took an average of ∼2 min (see Table 7 of Supplementary File 1 for complete details). However, it does not guarantee an exact posterior (Ghahramani and Beal 1999). Thus, we provide a computationally efficient hybrid approach in which

- Step 1: Fit the model using the VGA-based approach.
- Step 2: Estimate the component indicator variable *Z_ng_* conditional on the parameter estimates from the VGA-based approach.
- Step 3: Using the parameter estimates from Step 1 as the initial values for the parameters and using the classification from Step 2, compute the MCMC-based expectation for the latent variable $\theta_{ng}$ and obtain the final estimates of the model parameters.

The hybrid approach comes with a substantial reduction in computational overhead compared to a traditional MCMC-EM but it can generate samples from the exact posterior distribution. Fitting such a model using the hybrid approach on the same set of datasets from Simulation setting 2 with N=1000,rp=6, and *G *=* *2 took on average just under 10 min. Complete details of computational times for all three simulation settings are provided in Table 7 of Supplementary File 1. When the primary goal is to detect the underlying clusters (which is the case for the real data analysis), the VGA-based approach is sufficient. However, when the primarily goal is posterior inference, we recommend the hybrid approach as it can better yield an exact posterior similar to the MCMC-EM approach but is computationally efficient.

Details on the convergence criteria, initialization, and parallel implementation for an MCMC-EM approach is provided in Supplementary File 3.

### 2.4 Identifiability

Model identifiability is vital to obtain unique and consistent parameter estimates. Identifiability of univariate and multivariate finite mixtures of normal distributions has been proved (Teicher 1963; Yakowitz and Spragins 1968). With regard to the mixtures of MVPLN distributions, the estimates for $\Phi_{g}$ and $\Omega_{g}$ are only unique up to a strictly positive constant. To eliminate identifiability issues, a constraint needs to be imposed, e.g. the trace of $\Omega_{g}$ can be set equal to *p*, the trace of $\Phi_{g}$ can be set equal to *r*, or the first diagonal element of $\Phi_{g}$ can be set equal to 1. The latter solution, which is used by Gallaugher and McNicholas (2018), is used for all analyses in this paper. To obtain final parameter estimates, the resulting $\Phi_{g}^{\left(t\right)}$ is divided by the first diagonal element of $\Phi_{g}^{\left(t\right)}$, and $\Omega_{g}^{\left(t\right)}$ is multiplied by the first diagonal element of $\Phi_{g}^{\left(t\right)}$.

### 2.5 Model selection and performance assessment

Four model selection criteria are offered, which include the Akaike information criterion (AIC; Akaike 1973), the Bayesian information criterion (BIC; Schwarz 1978), a variation of the AIC used by Bozdogan (1994) called AIC3, and the integrated completed likelihood (ICL; Biernacki et al. 2000). These criteria are given by $\mathrm{AIC}=-2\;\text{log}\;L(\vartheta^{\left(\text{MLE}\right)}|y)+2K,\;\mathrm{BIC}=2\;\text{log}\;L(\vartheta^{\left(\text{MLE}\right)}|y)-K\;\text{log}\;N,\;\mathrm{AIC}3=-2\;\text{log}\;L(\vartheta^{\left(\text{MLE}\right)}|y)+3K$, and $\mathrm{ICL}\approx \mathrm{BIC}+2\sum_{n=1}^{N}\sum_{g=1}^{G}\text{MAP}\{{\hat{z}}_{ng}^{\left(t\right)}\}\;\text{log}\;{\hat{z}}_{ng}^{\left(t\right)}$, respectively, where $L(\vartheta^{\left(\text{MLE}\right)}|y)$ represents maximized log-likelihood, $\vartheta^{\left(\text{MLE}\right)}$ is the maximum likelihood estimate of the model parameters ϑ, *N* is the number of observations, and $\text{MAP}\{{\hat{z}}_{ng}^{\left(t\right)}\}$ is the maximum a posteriori classification given ${\hat{z}}_{ng}^{\left(t\right)}$.

In situations where the true classes are known but, for clustering purposes, are ignored, the adjusted Rand index (ARI; Hubert and Arabie 1985) can be used for performance assessment. The ARI takes a value 1 under perfect class agreement and has expected value 0 under random classification.

## 3 Results

### 3.1 Simulations

Simulation studies were conducted to illustrate the ability to recover the true underlying parameters for the mixtures of MVPLN algorithm. For Simulation 1, datasets with *G *=* *1 component were generated with *N *=* *1000 observations, *r *=* *2 and *p *=* *3. For Simulation 2, datasets with *G *=* *2 components and $\pi_{1}=0.79$ were generated with *N *=* *1000 observations, *r *=* *2 and *p *=* *3. For Simulation 3, datasets with *G *=* *2 components and $\pi_{1}=0.6$ were generated with *N *=* *1000 observations, *r *=* *2 and *p *=* *3. Further, only diagonal covariance structures for both $\Phi_{g}$ and $\Omega_{g}$ were considered in Simulation 3. Each of the simulation settings consisted of 25 different datasets. The count range in the simulated datasets closely represented the range observed in the RNA-seq data (Freixas-Coutin et al. 2017). The covariance matrices $\Phi_{g}$ and $\Omega_{g}$ for each setting are generated using the clusterGeneration package (Qiu and Joe 2015). Initialization of *z_ng_* was done using one hundred different runs of the *k*-means algorithm. Clustering was performed on each dataset for values G=1,…,5. We also compared the performance of our approach on count datasets generated from competitive models. For Simulation 4, we generated 25 datasets from a mixture of six independent Poisson distributions with *G *=* *2 components, $\pi_{1}=0.45$ and *N *=* *1000 and we analyzed the data as 2 × 3 matrix when using mixtures of MVPLN distributions. For Simulation 5, we generated 25 datasets from a mixture of six independent negative binomial distributions with *G *=* *2 components, $\pi_{1}=0.79$ and *N *=* *2000 and we analyzed the data as 2 × 3 matrix when using mixtures of MVPLN distributions. For Simulation 6, to show performance on a dataset with similar number of components to real data, we generated 25 datasets from a mixture of MVPLN distributions with *G *=* *8 components. We set *N *=* *1500, *r *=* *2, *p *=* *3, and $\pi_{1}=\cdots \pi_{8}=0.125$.

Comparative studies were also conducted on datasets from all five simulation settings. Because no comparable methods capable of clustering three-way count data were found in the current literature, datasets from Simulations 1, 2, 3, and 6 were vectorized and analyzed with clustering methods designed for two-way data. For comparison purpose, a model-based clustering technique for count data, HTSCluster (Rau et al. 2011, 2015), and a distance-based method, *k*-means clustering (MacQueen 1967) were used. For HTSCluster, initialization and clustering ranges were same as those used for mixtures of MVPLN algorithm. In a classification EM framework (CEM; Celeux and Govaert 1992), *k*-means has been shown to be equivalent to an isotropic Gaussian mixture model with equal variance across all components and with equal mixing proportions. Here, we fitted an equal variance isotropic Gaussian mixture model using R package mclust (Scrucca et al. 2016) on the log-transformed data which would be similar to fitting a fuzzy version of the *k*-means on the latent space and allow us to compute ICL that relies on the estimated soft $\hat{Z}$. We then utilized model selection criteria to select the optimal number of components.

The clustering results along with ARI values of our proposed method and other comparative methods are provided in Table 1. As evident in Table 1, in all six simulations, our proposed approach was able to recover the underlying cluster in all 25 datasets using both BIC and ICL, including when the Simulations 4 and 5, where the datasets were generated from mixtures of Poisson and negative binomial distributions. However, overfitting was evident with AIC and AIC3 as in various datasets, it favored models with larger number of components (i.e. *G *=* *3, 4, and 5 for Simulations 1–5 and *G *=* *9 and *G *=* *10 for Simulation 6). The AIC and AIC3 penalize log-likelihood only for the number of free parameters in the model and are constant with respect to the sample size. When the number of observations is large, these model selection criteria tend to favor more complex models and are known to overestimate number of components (Shibata 1976; Katz 1981). Note that we only provide the ARI values from the VGA approach. The ARI from the hybrid approach is the same as that from the VGA approach because the cluster membership indicator variable is determined in the VGA step in the hybrid approach. While we provide the estimated parameters for using MCMC approach, due to the extreme computational cost that comes with fitting these models, we only fitted the model with correct number of components, and thus, do not provide the summary in Table 1. For each simulation setting, we also provide a measure of cluster separation. For each simulated dataset, we computed the normalized separation index, I∈[0,1], by Hennig (2019) using the R package fpc (Hennig 2020), where higher values indicate that clusters have good separation. As Euclidean distance is used as a distance measure for computing the separation index, and we computed the separation index using the log-transformed data, which would be equivalent to assessing separation in the latent space. The average values of the separation index along with the standard deviation are provided in Table 1. The parameter estimation results for $M_{g},\;\Phi_{g}$, and $\Omega_{g}$ via the mixtures of MVPLN algorithm for Simulations 1–3 using all three approaches are summarized in Supplementary File 1. As can be seen through the simulations, all three approaches can recover the parameter estimates very well. However, there is a slight increase in the precision of these estimations with the hybrid approach. Given the large number of groups, for Simulation 6, the Frobenius norm of the difference between the estimated parameters and true values of the parameters are provided in Supplementary File 1. In Table 7 of Supplementary File 1, we provide the computation time taken to fit the mixtures of MVPLN model with correct number of components using all three components. Although all three approaches provide comparable parameter estimates, the computational time taken to fit MVPLN using MCMC-based approach is substantially larger than for the VGA and the hybrid approaches as seen in Table 7 of Supplementary File 1. Overall, the simulation experiments illustrated that our approach for parameter estimation (Section 2.3) is effective at parameter recovery for the mixtures of MVPLN distributions.

**Table 1.** Mean of normalized cluster separation index (standard deviation) for each simulation setting and the number of clusters selected (average ARI, standard deviation) from all 25 datasets for each simulation setting using different model selection criteria.

Method	Simulation setting	Mean of normalized separation index (SD)	BIC	ICL	AIC	AIC3
Mix. of MVPLN	1	–	1 (1.00, 0.00)	1 (1.00, 0.00)	1, 2 (0.88, 0.33)	1 (1.00, 0.00)
(VGA-based	2	0.44 (0.02)	2 (1.00, 0.00)	2 (1.00, 0.00)	2, 3, 4, 5 (0.89, 0.22)	2 (1.00, 0.00)
approach)	3	0.37 (0.02)	2 (1.00, 0.00)	2 (1.00, 0.00)	2, 3, 4, 5 (0.86, 0.21)	2, 3, 4 (0.96, 0.12)
	4	0.69 (0.01)	2 (1.00, 0.00)	2 (1.00, 0.00)	2 (1.00, 0.00)	2 (1.00, 0.00)
	5	0.44 (0.02)	2 (1.00, 0.00)	2 (1.00, 0.00)	2, 5 (0.99, 0.06)	2 (1.00, 0.00)
	6	0.14 (0.01)	8 (0.99, 0.00)	8 (0.99, 0.00)	8, 9, 10 (0.99, 0.01)	8, 9 (0.99, 0.01)
HTSCluster	1	–	5 (0.00, 0.00)	5 (0.00, 0.00)	5 (0.00, 0.00)	5 (0.00, 0.00)
	2	0.44 (0.02)	5 (−0.00, 0.00)	5 (−0.00, 0.00)	5 (−0.00, 0.00)	5 (−0.00, 0.00)
	3	0.37 (0.02)	5 (0.00, 0.01)	5 (0.00, 0.01)	5 (0.00, 0.01)	5 (0.00, 0.01)
	4	0.69 (0.01)	2 (1.00, 0.00)	2 (1.00, 0.00)	2, 3, 4 (0.97, 0.06)	2, 3, 4 (0.97, 0.06)
	5	0.44 (0.02)	5 (0.22, 0.01)	5 (0.22, 0.01)	5 (0.22, 0.01)	5 (0.22, 0.01)
	6	0.14 (0.01)	10 (0.03, 0.13)	10 (0.03, 0.13)	10 (0.03, 0.13)	10 (0.03, 0.13)
Fuzzy-version of	1	–	5 (0.00, 0.00)	4,5 (0.00, 0.00)	5 (0.00, 0.00)	5 (0.00, 0.00)
*k*-means	2	0.44 (0.02)	5 (0.24, 0.01)	5 (0.24, 0.01)	5 (0.24, 0.01)	5 (0.24, 0.01)
	3	0.37 (0.02)	3, 4, 5 (0.55, 0.06)	2, 3, 4, 5 (0.81, 0.23)	4, 5 (0.54, 0.04)	4, 5 (0.54, 0.04)
	4	0.69 (0.01)	2 (1.00, 0.00)	2 (1.00, 0.00)	2, 4, 5 (0.54, 0.12)	2, 4, 5 (0.54, 0.12)
	5	0.44 (0.02)	2 (1.00, 0.00)	2 (1.00, 0.00)	2, 3, 4, 5 (0.82, 0.27)	2, 3, 4, 5 (0.82, 0.27)
	6	0.14 (0.01)	8, 9, 10 (0.93, 0.03)	8, 9, 10 (0.96, 0.02)	10 (0.90, 0.01)	10 (0.90, 0.01)

In the two simulation settings where the datasets were generated from mixtures of independent Poisson distributions and mixtures of independent negative binomial distributions (Simulation 4 and 5), our approach was able to recover the underlying cluster structure and estimate the mean and variances of samples fairly well. With regard to HTSCluster, a model with *G *=* *5, the highest value for *G* considered, was selected for in Simulations 1, 2, 3, and 5 and a model with *G *=* *10, the highest value for *G* considered, was selected for in Simulation 6. Furthermore, the ARI values were low across these simulation settings, indicating that observations were not assigned to the correct clusters. However, in simulation setting 4 where the datasets are generated from mixtures of independent Poisson distribution, HTSCluster is able to recover the underlying cluster structure in all 25 datasets using BIC and ICL with perfect classification. It is worth noting that several studies have demonstrated that in presence of biological variations, the observed variability in the RNA-seq data is greater than what is predicted by the Poisson model (Gao et al. 2010). Thus, the negative binomial distribution (a hierarchical Poisson model similar to the univariate version of the MPLN model) has emerged as the most predominantly used distribution of choice for univariate analysis involving RNA-seq data (Gao et al. 2010). For *k*-means clustering, the average ARI values were also low for Simulations 1–3 but the value for Simulation 6 was high and perfect classification was observed in Simulations 4 and 5 using BIC and ICL.

### 3.2 Clustering transcriptome data

To illustrate the applicability of mixtures of MVPLN distributions for detecting the underlying cluster structure, the VGA-based approach was applied to an RNA-seq dataset. Typically, only a subset of genes from the experiment are used for cluster analysis, in order to reduce noise. For this analysis, only the differentially expressed genes were used for clustering. Freixas-Coutin et al. (2017) used RNA-seq to monitor the transcriptional dynamics in the seed coats of darkening and nondarkening cranberry beans (*Phaseolus vulgaris*) at three developmental stages: early, intermediate and mature. The aim of the study was to evaluate if the changes in the seed coat transcriptome were associated with proanthocyanidin levels as a function of seed development in cranberry beans. The RNA-seq data are available on the National Center for Biotechnology Information (NCBI) Sequence Read Archive (SRA) under the BioProject PRJNA380220.

**Figure 4.**
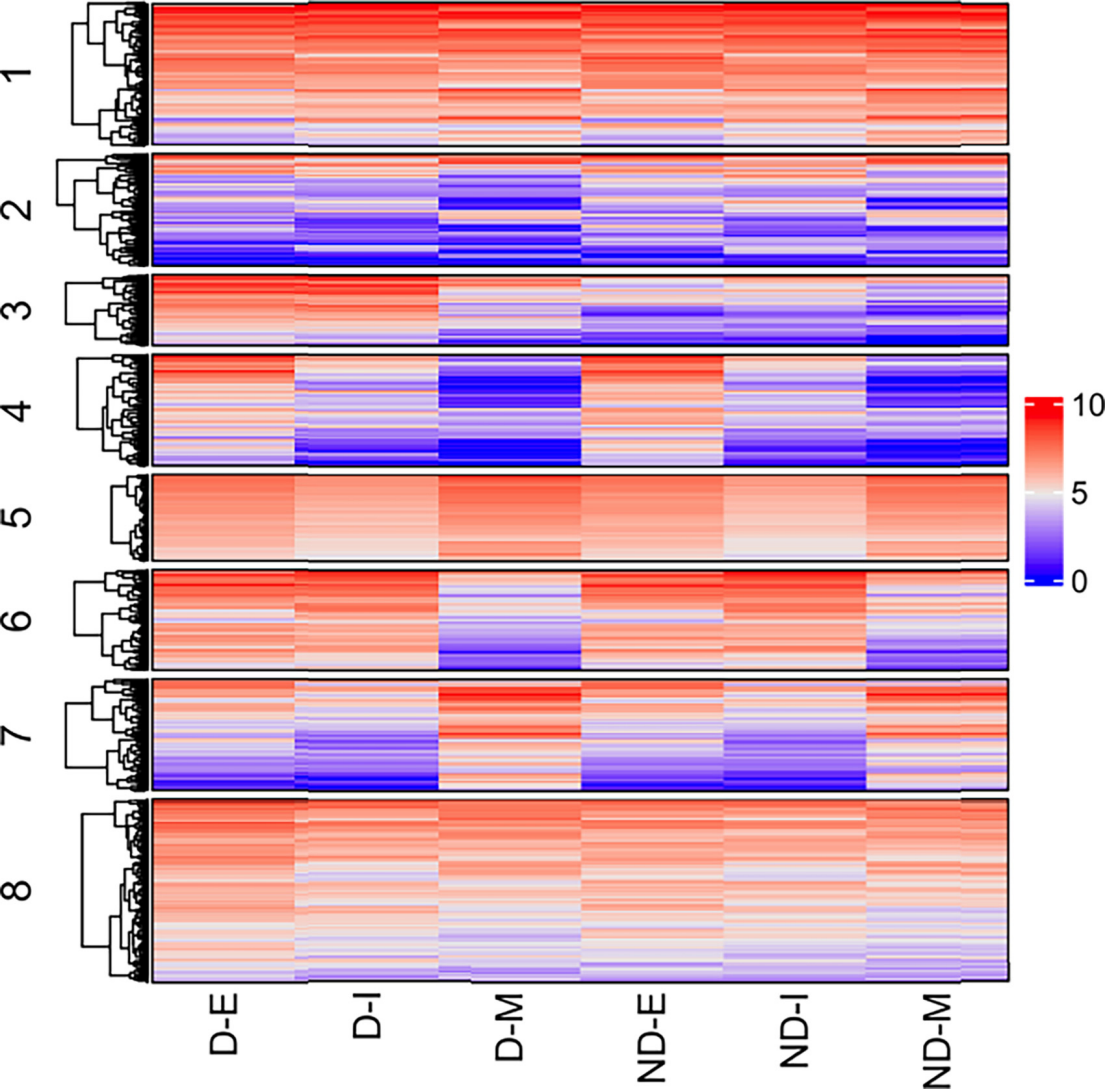
Heatmap showing, for Cluster 1 through Cluster 8, log-transformed gene expression patterns for the *G *=* *8 model selected by both BIC and ICL for the cranberry bean RNA-seq dataset. The red and blue colors represent the log-transformed expression levels, where red represents high expression and blue represents low expression. The rows represent the genes and the columns represent samples involved in the RNA-seq study. Respectively, the samples are D-E: darkening early, D-I: darkening intermediate, D-M: darkening mature, ND-E: nondarkening early, ND-I: nondarkening intermediate, and ND-M: nondarkening mature cranberry bean.

The study identified 1336 differentially expressed genes, which were used for clustering. The raw read counts for genes were obtained from Binary Alignment/Map files using samtools (Li et al. 2009) and HTSeq (Anders et al. 2015). The median value from the three replicates per each developmental stage was used. Each observation or gene (the unit) was structured as a 2 × 3 matrix, such that it contained counts for the two varieties (the variables): darkening and nondarkening, across the three developmental stages (the occasions): early, intermediate and mature. On the three-way data of dimensions 1336×2×3, a clustering range of G=1,…,10 was considered using *k*-means initialization (100 runs). Furthermore, we repeated the analysis 10 times. Since BIC and ICL both performed well in recovering the underlying cluster structure in the simulated data, here we also used BIC and ICL for model selection. Both BIC and ICL selected a model with *G *=* *8. In this model, Clusters 1–8 were composed of 206 (15.4%), 163 (12.2%), 104 (7.8%), 162 (12.1%), 126 (9.4%), 147 (11.0%), 162 (12.1%), and 266 (19.9%) genes, respectively. See Supplementary File 2 for gene composition of each cluster. Expression patterns across the clusters were visualized using a heatmap. The log-transformed expression patterns of the clusters are illustrated using the heatmap in Fig. 4.

**Figure 5.**
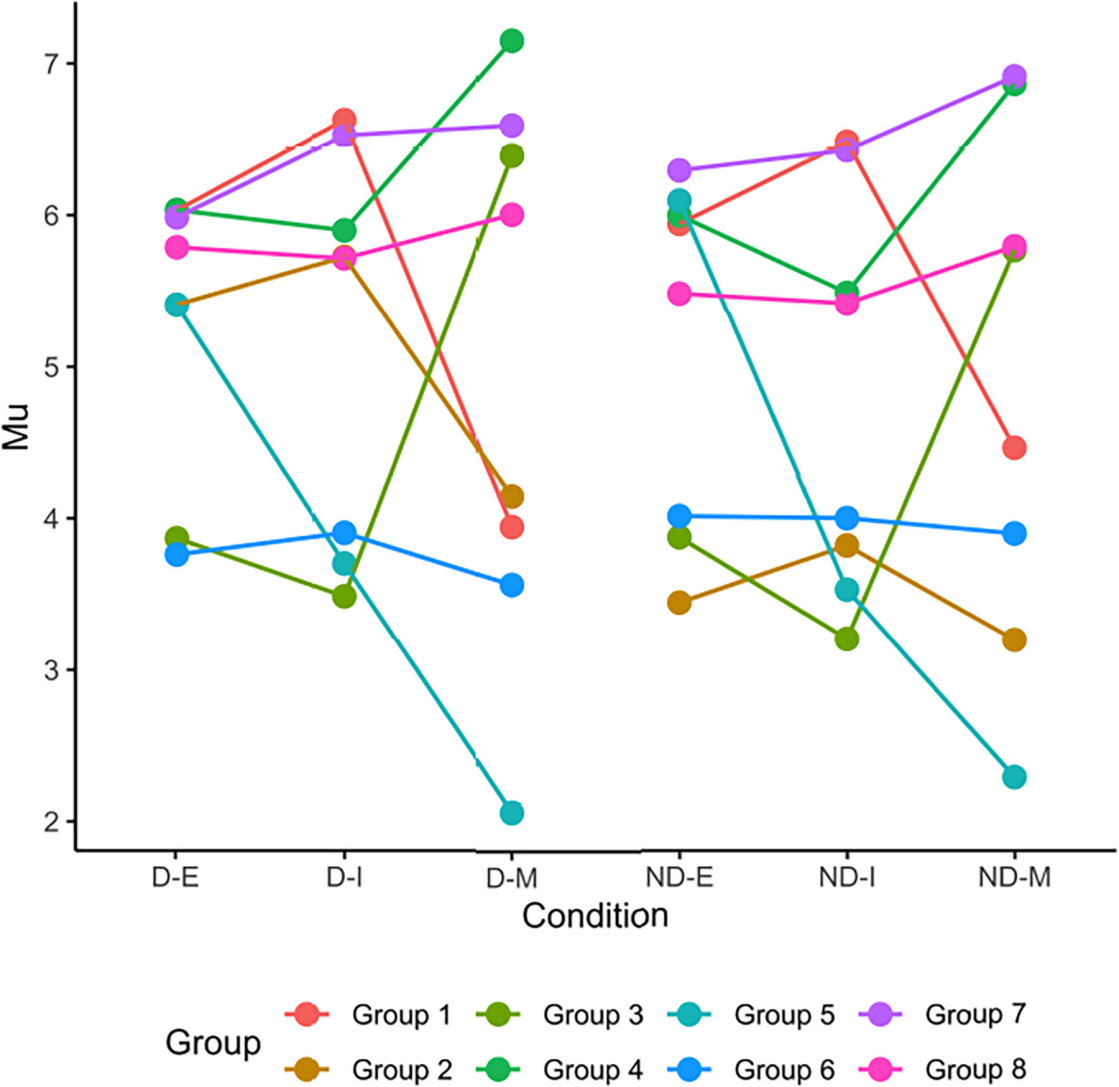
Visualization of the cluster-specific $\mu_{g}$ which relates to the mean trends of the expression levels of genes in Clusters 1–8.

In simulation studies where the true labels are available and ARI of true and predicted class label can be used to assess clustering performance. Because no true labels are available in real data, we use visualization of the data using heatmap to assess the trends in the clusters obtained on the transcriptome data in Fig. 4. We also visualize the cluster-specific *μ_g_* which relates to mean trends of the expression levels of genes in different clusters in Fig. 5. As evident in Figs 4 and 5, each cluster has its distinctive expression signatures. In some clusters (for example, Cluster 4), the means of the gene expression signatures are similar between the darkening and nondarkening beans but their mean expression pattern varies between developmental stages; whereas, in Cluster 8, the means of the gene expression signatures are similar across the developmental stages but varies slightly between the darkening and nondarkening beans. On the other hand, in Cluster 3, the mean gene expression signatures vary across both the developmental stages as well as between darkening and nondarkening beans.

For all simulation and transcriptome data analyses, the normalization factors representing library sizes for samples were obtained using the trimmed mean of *M* values from calcNormFactors function of edgeR package (Robinson and Oshlack 2010; McCarthy et al. 2012).

A table of mathematical notation is provided in Supplementary File 4 and a table of abbreviations is provided in Supplementary File 5.

## 4 Discussion

A mixture of MVPLN distributions is introduced for clustering three-way count data, targeted at expression data arising from RNA-seq experiments. This is the first use of a mixture of MVPLN distributions for clustering within the literature. By allowing for a direct analysis of three-way data structures, matrix variate distributions permit the estimation of correlations within and between variables and occasions. This makes them very attractive for analyzing matrix data in the context of clustering. Further, by considering a matrix variate structure, the number of free covariance parameters to be estimated is greatly reduced under high dimensional settings. Herein, three different parameter estimation frameworks are proposed: an approach based on MCMC, one based on VGA, and one based on a hybrid approach. When posterior inference is of interest, the MCMC-based approach is favorable but it can be computationally intensive. On the other hand, the VGA-based approach only approximates the posterior distribution that relies on approximation but it is computationally efficient. Therefore, here we also propose a hybrid approach that is computationally efficient and it samples from the true posterior. Through simulation studies, we show that the VGA approach provides good clustering performance even when the datasets are generated from mixtures of other discrete distributions.

Using simulated data, it was illustrated that the algorithm for mixtures of MVPLN distributions is effective and returned favorable clustering results. The transcriptome data analysis showed the applicability of the mixture model-based clustering method on RNA-seq count data. A possible future direction of this work would be to make use of subspace clustering methods and to develop the matrix variate factor analyzers model. This would permit clustering of data in low-dimensional subspaces as high-dimensional RNA-seq datasets become frequent. Another path is to consider restrictions on the matrices $\Phi_{g}$ and $\Omega_{g}$, as done by Viroli (2011). Also, constraints on $\Phi_{g}$ similar to those introduced by McNicholas and Murphy (2010), and used by Anderlucci and Viroli (2015), could be beneficial when analyzing longitudinal RNA-seq data.

## Supplementary Material

btad167_Supplementary_Data

## Data Availability

The RNA-seq data used in the manuscript are publicly available on the National Center for Biotechnology Information (NCBI) Sequence Read Archive (SRA) under the BioProject PRJNA380220. The GitHub R package for this work is available at https://github.com/anjalisilva/mixMVPLN.
