# Supplementary material for: Finite mixtures of matrix variate Poisson-log normal distributions for three-way count data
Source: Bioinformatics. 2023 Apr 5;39(5):btad167. doi: 10.1093/bioinformatics/btad167 (PMC10159656; doi:10.1093/bioinformatics/btad167)
Supplement: btad167_Supplementary_Data [file btad167_supplementary_data.zip › Supplementary File/Supplementary_File_5.pdf]

1 Abbreviations

Table 1: List of abbreviations

Abbreviation	Full name
AIC	Akaike information criterion
AIC3	Bozdogan Akaike information criterion
ARI	adjusted Rand index
BIC	Bayesian information criterion
CPU	central processing unit
D	darkening
E	early
E-step	expectation step
GO	gene ontology
HPC	high performance computing
I	intermediate
ICL	integrated completed likelihood
M	mature
M-step	maximization step
MAP	maximum *a posteriori* probability
MCMC-EM	Markov chain Monte Carlo expectation-maximization
MPLN	multivariate Poisson-log normal
MVPLN	matrix variate Poisson-log normal
NCBI	National Center for Biotechnology Information
ND	non-darkening
RAM	random-access memory
RNA-seq	RNA sequencing
SRA	sequence read archive
TMM	trimmed mean of M values
P. vulgaris	*Phaseolus vulgaris*
