# Supplementary material for: Finite mixtures of matrix variate Poisson-log normal distributions for three-way count data
Source: Bioinformatics. 2023 Apr 5;39(5):btad167. doi: 10.1093/bioinformatics/btad167 (PMC10159656; doi:10.1093/bioinformatics/btad167)
Supplement: btad167_Supplementary_Data [file btad167_supplementary_data.zip › Supplementary File/Supplementary_File_4.pdf]

1 Notations

Table 1: Description of notations used

Notation	Description
$\mathbf{T}$	a random matrix
$\mathbf{Y}$	a random matrix
$\text{vec}(\mathbf{Y})$	a vectorized matrix of $\mathbf{Y}$
$\mathbf{I}$	an identity matrix
$\mathbf{M}$	$r \times p$ matrix of means
$\mathbf{\Phi}$	$r \times r$ covariance matrix containing the variances and covariances between r occasions
$\mathbf{\Omega}$	$p \times p$ covariance matrix containing the variance and covariances of the p variables
$\mathbf{\Sigma}$	covariance matrix separable in the form $\mathbf{\Sigma} = \mathbf{\Phi} \otimes \mathbf{\Omega}$
k	number of responses/conditions (variables), such that $k = 1, \ldots, p$
p	maximum number of responses/conditions
i	number of occasions/replicates (layers), such that $i = 1, \ldots, r$
r	maximum number of occasions/replicates
n	number of units, such that $n = 1, \ldots, N$
N	maximum number of units
n_g	number of observations in each cluster g
c	dimensionality/number of samples, such that $c = 1, \ldots, rp$
rp	maximum size of dimensionality/samples
g	number of components/clusters, such that $g = 1, \ldots, G$
G	maximum number of components/clusters
π	mixing proportions
$\boldsymbol{\vartheta}$	vector of model parameters
$\mathscr{P}$	Poisson distribution
$\mathcal{N}$	a normal distribution
s_c	a known constant for library size of a given sample c
$\text{vec}(\mathbf{s})$	a vector of library sizes
$\boldsymbol{\theta}^{(f)}$	random sample simulated via `RStan` package for each iteration
$\otimes$	Kronecker product
$\mathbb{E}$	expected value
z	indicator variable

f	number of iterations, such that $f = 1, \ldots, B$
B	number total iterations
W	number of iterations used in `RStan` for parameter estimation, after discarding values from initial iterations to minimize bias
$\mathcal{Q}$	expected value of the complete-data log-likelihood
C	a constant with respect to some parameters
l_c	complete-data log-likelihood
$\boldsymbol{\Theta}$	vector of model parameters and mixing proportions
tr	trace
MAP	maximum *a posteriori* classification
K	number of free parameters
exp	exponential function
