# Supplementary material for: Finite mixtures of matrix variate Poisson-log normal distributions for three-way count data
Source: Bioinformatics. 2023 Apr 5;39(5):btad167. doi: 10.1093/bioinformatics/btad167 (PMC10159656; doi:10.1093/bioinformatics/btad167)
Supplement: btad167_Supplementary_Data [file btad167_supplementary_data.zip › Supplementary File/Supplementary_File_1.pdf]

1 Parameter Estimation Results

1.1 Simulation Setting 1

Table 1: Model parameters ($\boldsymbol{\vartheta}_g$) as well as means and standard deviations of the associated parameter estimates from the 25 datasets for Simulation 1.

(a) Using MCMC based approach.

$\boldsymbol{\vartheta}_g$	True value	Means	Standard deviations
$\mathbf{M}_1$	$\begin{bmatrix} 6.00 & 5.50 & 6.00 \\ 6.00 & 5.50 & 6.00 \end{bmatrix}$	$\begin{bmatrix} 6.03 & 5.67 & 6.13 \\ 5.80 & 5.48 & 5.91 \end{bmatrix}$	$\begin{bmatrix} 0.07 & 0.07 & 0.06 \\ 0.11 & 0.07 & 0.08 \end{bmatrix}$
$\boldsymbol{\Phi}_1$	$\begin{bmatrix} 1.00 & -0.55 \\ -0.55 & 1.27 \end{bmatrix}$	$\begin{bmatrix} 1.00 & -0.55 \\ -0.55 & 1.30 \end{bmatrix}$	$\begin{bmatrix} 0.00 & 0.02 \\ 0.02 & 0.04 \end{bmatrix}$
$\boldsymbol{\Omega}_1$	$\begin{bmatrix} 1.66 & -0.61 & 0.77 \\ -0.61 & 1.46 & 0.17 \\ 0.77 & 0.17 & 1.44 \end{bmatrix}$	$\begin{bmatrix} 1.65 & -0.60 & 0.78 \\ -0.60 & 1.43 & 0.17 \\ 0.78 & 0.17 & 1.45 \end{bmatrix}$	$\begin{bmatrix} 0.06 & 0.05 & 0.04 \\ 0.05 & 0.05 & 0.05 \\ 0.04 & 0.05 & 0.06 \end{bmatrix}$

(b) Using VGA approach

$\boldsymbol{\vartheta}_g$	True value	Means	Standard deviations
$\mathbf{M}_1$	$\begin{bmatrix} 6.00 & 5.50 & 6.00 \\ 6.00 & 5.50 & 6.00 \end{bmatrix}$	$\begin{bmatrix} 6.03 & 5.66 & 6.13 \\ 5.80 & 5.47 & 5.92 \end{bmatrix}$	$\begin{bmatrix} 0.07 & 0.07 & 0.06 \\ 0.11 & 0.07 & 0.08 \end{bmatrix}$
$\boldsymbol{\Phi}_1$	$\begin{bmatrix} 1.00 & -0.55 \\ -0.55 & 1.27 \end{bmatrix}$	$\begin{bmatrix} 1.00 & -0.56 \\ -0.56 & 1.29 \end{bmatrix}$	$\begin{bmatrix} 0.00 & 0.01 \\ 0.01 & 0.04 \end{bmatrix}$
$\boldsymbol{\Omega}_1$	$\begin{bmatrix} 1.66 & -0.61 & 0.77 \\ -0.61 & 1.46 & 0.17 \\ 0.77 & 0.17 & 1.44 \end{bmatrix}$	$\begin{bmatrix} 1.66 & -0.61 & 0.78 \\ -0.61 & 1.45 & 0.17 \\ 0.78 & 0.17 & 1.44 \end{bmatrix}$	$\begin{bmatrix} 0.06 & 0.05 & 0.04 \\ 0.05 & 0.06 & 0.05 \\ 0.04 & 0.05 & 0.05 \end{bmatrix}$

(c) Using hybrid approach (i.e., VGA approach followed by MCMC approach)

$\boldsymbol{\vartheta}_g$	True value	Means	Standard deviations
$\mathbf{M}_1$	$\begin{bmatrix} 6.00 & 5.50 & 6.00 \\ 6.00 & 5.50 & 6.00 \end{bmatrix}$	$\begin{bmatrix} 6.00 & 5.52 & 6.01 \\ 5.98 & 5.51 & 5.99 \end{bmatrix}$	$\begin{bmatrix} 0.06 & 0.04 & 0.05 \\ 0.05 & 0.04 & 0.05 \end{bmatrix}$
$\boldsymbol{\Phi}_1$	$\begin{bmatrix} 1.00 & -0.55 \\ -0.55 & 1.27 \end{bmatrix}$	$\begin{bmatrix} 1.00 & -0.55 \\ -0.55 & 1.29 \end{bmatrix}$	$\begin{bmatrix} 0.00 & 0.02 \\ 0.02 & 0.04 \end{bmatrix}$
$\boldsymbol{\Omega}_1$	$\begin{bmatrix} 1.66 & -0.61 & 0.77 \\ -0.61 & 1.46 & 0.17 \\ 0.77 & 0.17 & 1.44 \end{bmatrix}$	$\begin{bmatrix} 1.67 & -0.61 & 0.78 \\ -0.61 & 1.45 & 0.17 \\ 0.78 & 0.17 & 1.45 \end{bmatrix}$	$\begin{bmatrix} 0.07 & 0.05 & 0.04 \\ 0.05 & 0.06 & 0.05 \\ 0.04 & 0.05 & 0.06 \end{bmatrix}$

1.2 Simulation Setting 2

Table 2: Model parameters ($\boldsymbol{\vartheta}_g$) as well as means and standard deviations of the associated parameter estimates from the 25 datasets for Simulation 2.

(a) Using MCMC based approach

$\boldsymbol{\vartheta}_g$	True value	Means	Standard deviations
$\mathbf{M}_1$	$\begin{bmatrix} 6.00 & 6.00 & 6.00 \\ 6.00 & 6.00 & 6.00 \end{bmatrix}$	$\begin{bmatrix} 6.06 & 5.75 & 6.18 \\ 5.90 & 6.18 & 5.94 \end{bmatrix}$	$\begin{bmatrix} 0.08 & 0.06 & 0.08 \\ 0.09 & 0.07 & 0.08 \end{bmatrix}$
$\mathbf{M}_2$	$\begin{bmatrix} 1.00 & 1.00 & 1.00 \\ 1.00 & 1.00 & 1.00 \end{bmatrix}$	$\begin{bmatrix} 1.05 & 0.84 & 1.09 \\ 0.97 & 1.14 & 0.97 \end{bmatrix}$	$\begin{bmatrix} 0.12 & 0.08 & 0.11 \\ 0.08 & 0.08 & 0.13 \end{bmatrix}$
$\mathbf{\Phi}_1$	$\begin{bmatrix} 1.00 & -0.62 \\ -0.62 & 1.40 \end{bmatrix}$	$\begin{bmatrix} 1.00 & -0.63 \\ -0.63 & 1.40 \end{bmatrix}$	$\begin{bmatrix} 0.00 & 0.02 \\ 0.02 & 0.06 \end{bmatrix}$
$\mathbf{\Phi}_2$	$\begin{bmatrix} 1.00 & 0.00 \\ 0.00 & 1.08 \end{bmatrix}$	$\begin{bmatrix} 1.00 & -0.01 \\ -0.01 & 0.89 \end{bmatrix}$	$\begin{bmatrix} 0.00 & 0.05 \\ 0.05 & 0.13 \end{bmatrix}$
$\mathbf{\Omega}_1$	$\begin{bmatrix} 1.66 & -0.61 & 0.77 \\ -0.61 & 1.46 & 0.17 \\ 0.77 & 0.17 & 1.44 \end{bmatrix}$	$\begin{bmatrix} 1.18 & -0.44 & 0.55 \\ -0.44 & 1.05 & 0.12 \\ 0.55 & 0.12 & 1.02 \end{bmatrix}$	$\begin{bmatrix} 0.50 & 0.19 & 0.24 \\ 0.19 & 0.44 & 0.06 \\ 0.24 & 0.06 & 0.43 \end{bmatrix}$
$\mathbf{\Omega}_2$	$\begin{bmatrix} 1.00 & 0.00 & 0.00 \\ 0.00 & 1.04 & 0.00 \\ 0.00 & 0.00 & 1.10 \end{bmatrix}$	$\begin{bmatrix} 0.89 & -0.01 & 0.01 \\ -0.01 & 1.05 & 0.01 \\ 0.01 & 0.01 & 1.08 \end{bmatrix}$	$\begin{bmatrix} 0.13 & 0.06 & 0.06 \\ 0.06 & 0.17 & 0.07 \\ 0.06 & 0.07 & 0.22 \end{bmatrix}$

(b) Using VGA approach

$\boldsymbol{\vartheta}_g$	True value	Means	Standard deviations
$\mathbf{M}_1$	$\begin{bmatrix} 6.00 & 6.00 & 6.00 \\ 6.00 & 6.00 & 6.00 \end{bmatrix}$	$\begin{bmatrix} 6.01 & 6.02 & 6.03 \\ 5.98 & 5.99 & 6.00 \end{bmatrix}$	$\begin{bmatrix} 0.06 & 0.06 & 0.04 \\ 0.07 & 0.07 & 0.06 \end{bmatrix}$
$\mathbf{M}_2$	$\begin{bmatrix} 1.00 & 1.00 & 1.00 \\ 1.00 & 1.00 & 1.00 \end{bmatrix}$	$\begin{bmatrix} 1.15 & 1.14 & 1.15 \\ 1.15 & 1.15 & 1.17 \end{bmatrix}$	$\begin{bmatrix} 0.08 & 0.09 & 0.11 \\ 0.09 & 0.07 & 0.09 \end{bmatrix}$
$\mathbf{\Phi}_1$	$\begin{bmatrix} 1.00 & -0.62 \\ -0.62 & 1.40 \end{bmatrix}$	$\begin{bmatrix} 1.00 & -0.63 \\ -0.63 & 1.41 \end{bmatrix}$	$\begin{bmatrix} 0.00 & 0.02 \\ 0.02 & 0.05 \end{bmatrix}$
$\mathbf{\Phi}_2$	$\begin{bmatrix} 1.00 & 0.00 \\ 0.00 & 1.08 \end{bmatrix}$	$\begin{bmatrix} 1.00 & 0.01 \\ 0.01 & 0.94 \end{bmatrix}$	$\begin{bmatrix} 0.00 & 0.08 \\ 0.08 & 0.18 \end{bmatrix}$
$\mathbf{\Omega}_1$	$\begin{bmatrix} 1.66 & -0.61 & 0.77 \\ -0.61 & 1.46 & 0.17 \\ 0.77 & 0.17 & 1.44 \end{bmatrix}$	$\begin{bmatrix} 1.66 & -0.62 & 0.76 \\ -0.66 & 1.46 & 0.18 \\ 0.76 & 0.18 & 1.43 \end{bmatrix}$	$\begin{bmatrix} 0.08 & 0.04 & 0.06 \\ 0.04 & 0.06 & 0.04 \\ 0.06 & 0.04 & 0.08 \end{bmatrix}$
$\mathbf{\Omega}_2$	$\begin{bmatrix} 1.00 & 0.00 & 0.00 \\ 0.00 & 1.04 & 0.00 \\ 0.00 & 0.00 & 1.10 \end{bmatrix}$	$\begin{bmatrix} 0.59 & 0.00 & 0.01 \\ 0.00 & 0.70 & 0.02 \\ 0.01 & 0.02 & 0.74 \end{bmatrix}$	$\begin{bmatrix} 0.09 & 0.07 & 0.07 \\ 0.07 & 0.10 & 0.08 \\ 0.07 & 0.08 & 0.12 \end{bmatrix}$

(c) Using hybrid approach (i.e., VGA approach followed by MCMC approach)

$\boldsymbol{\vartheta}_g$	True value	Means	Standard deviations
$\mathbf{M}_1$	$\begin{bmatrix} 6.00 & 6.00 & 6.00 \\ 6.00 & 6.00 & 6.00 \end{bmatrix}$	$\begin{bmatrix} 6.01 & 6.02 & 6.02 \\ 5.98 & 5.99 & 5.99 \end{bmatrix}$	$\begin{bmatrix} 0.06 & 0.06 & 0.04 \\ 0.07 & 0.07 & 0.06 \end{bmatrix}$
$\mathbf{M}_2$	$\begin{bmatrix} 1.00 & 1.00 & 1.00 \\ 1.00 & 1.00 & 1.00 \end{bmatrix}$	$\begin{bmatrix} 1.11 & 1.11 & 1.11 \\ 1.12 & 1.12 & 1.13 \end{bmatrix}$	$\begin{bmatrix} 0.08 & 0.09 & 0.11 \\ 0.09 & 0.07 & 0.09 \end{bmatrix}$
$\mathbf{\Phi}_1$	$\begin{bmatrix} 1.00 & -0.62 \\ -0.62 & 1.40 \end{bmatrix}$	$\begin{bmatrix} 1.00 & -0.63 \\ -0.63 & 1.40 \end{bmatrix}$	$\begin{bmatrix} 0.00 & 0.02 \\ 0.02 & 0.05 \end{bmatrix}$
$\mathbf{\Phi}_2$	$\begin{bmatrix} 1.00 & 0.00 \\ 0.00 & 1.08 \end{bmatrix}$	$\begin{bmatrix} 1.00 & 0.00 \\ 0.00 & 0.96 \end{bmatrix}$	$\begin{bmatrix} 0.00 & 0.07 \\ 0.07 & 0.18 \end{bmatrix}$
$\mathbf{\Omega}_1$	$\begin{bmatrix} 1.66 & -0.61 & 0.77 \\ -0.61 & 1.46 & 0.17 \\ 0.77 & 0.17 & 1.44 \end{bmatrix}$	$\begin{bmatrix} 1.68 & -0.63 & 0.77 \\ -0.63 & 1.48 & 0.18 \\ 0.77 & 0.18 & 1.45 \end{bmatrix}$	$\begin{bmatrix} 0.08 & 0.04 & 0.06 \\ 0.04 & 0.05 & 0.04 \\ 0.06 & 0.04 & 0.08 \end{bmatrix}$
$\mathbf{\Omega}_2$	$\begin{bmatrix} 1.00 & 0.00 & 0.00 \\ 0.00 & 1.04 & 0.00 \\ 0.00 & 0.00 & 1.10 \end{bmatrix}$	$\begin{bmatrix} 0.88 & 0.00 & 0.01 \\ 0.00 & 1.03 & 0.02 \\ 0.01 & 0.02 & 1.08 \end{bmatrix}$	$\begin{bmatrix} 0.12 & 0.10 & 0.09 \\ 0.10 & 0.13 & 0.10 \\ 0.09 & 0.10 & 0.17 \end{bmatrix}$

1.3 Simulation Setting 3

Table 3: Model parameters ($\boldsymbol{\vartheta}_g$) as well as means and standard deviations of the associated parameter estimates from the 25 datasets for Simulation 3.

(a) Using MCMC based approach

$\boldsymbol{\vartheta}_g$	True value	Means	Standard deviations
$\mathbf{M}_1$	$\begin{bmatrix} 6.20 & 6.20 & 6.20 \\ 6.20 & 6.20 & 6.20 \end{bmatrix}$	$\begin{bmatrix} 6.26 & 5.91 & 6.38 \\ 6.11 & 6.42 & 6.14 \end{bmatrix}$	$\begin{bmatrix} 0.09 & 0.08 & 0.07 \\ 0.08 & 0.07 & 0.09 \end{bmatrix}$
$\mathbf{M}_2$	$\begin{bmatrix} 1.50 & 1.50 & 1.50 \\ 1.50 & 1.50 & 1.50 \end{bmatrix}$	$\begin{bmatrix} 1.55 & 1.35 & 1.60 \\ 1.52 & 1.69 & 1.44 \end{bmatrix}$	$\begin{bmatrix} 0.10 & 0.05 & 0.08 \\ 0.06 & 0.07 & 0.13 \end{bmatrix}$
$\mathbf{\Phi}_1$	$\begin{bmatrix} 1.00 & 0.00 \\ 0.00 & 1.00 \end{bmatrix}$	$\begin{bmatrix} 1.00 & -0.01 \\ -0.01 & 1.40 \end{bmatrix}$	$\begin{bmatrix} 0.00 & 0.03 \\ 0.03 & 0.07 \end{bmatrix}$
$\mathbf{\Phi}_2$	$\begin{bmatrix} 1.00 & 0.00 \\ 0.00 & 0.70 \end{bmatrix}$	$\begin{bmatrix} 1.00 & -0.00 \\ -0.00 & 0.56 \end{bmatrix}$	$\begin{bmatrix} 0.00 & 0.03 \\ 0.03 & 0.05 \end{bmatrix}$
$\mathbf{\Omega}_1$	$\begin{bmatrix} 1.67 & 0.00 & 0.00 \\ 0.00 & 1.46 & 0.00 \\ 0.00 & 0.00 & 1.44 \end{bmatrix}$	$\begin{bmatrix} 1.22 & -0.00 & -0.01 \\ -0.00 & 1.08 & 0.00 \\ -0.01 & 0.00 & 1.05 \end{bmatrix}$	$\begin{bmatrix} 0.49 & 0.03 & 0.03 \\ 0.03 & 0.43 & 0.04 \\ 0.03 & 0.04 & 0.43 \end{bmatrix}$
$\mathbf{\Omega}_2$	$\begin{bmatrix} 0.75 & 0.00 & 0.00 \\ 0.00 & 0.82 & 0.00 \\ 0.00 & 0.00 & 0.90 \end{bmatrix}$	$\begin{bmatrix} 0.83 & -0.01 & -0.00 \\ -0.01 & 1.04 & 0.00 \\ -0.00 & 0.00 & 1.17 \end{bmatrix}$	$\begin{bmatrix} 0.15 & 0.04 & 0.05 \\ 0.04 & 0.15 & 0.07 \\ 0.05 & 0.07 & 0.18 \end{bmatrix}$

(b) Using VGA approach

$\boldsymbol{\vartheta}_g$	True value	Means	Standard deviations
$\mathbf{M}_1$	$\begin{bmatrix} 6.20 & 6.20 & 6.20 \\ 6.20 & 6.20 & 6.20 \end{bmatrix}$	$\begin{bmatrix} 6.27 & 6.39 & 6.42 \\ 5.91 & 6.11 & 6.14 \end{bmatrix}$	$\begin{bmatrix} 0.09 & 0.07 & 0.07 \\ 0.08 & 0.08 & 0.09 \end{bmatrix}$
$\mathbf{M}_2$	$\begin{bmatrix} 1.50 & 1.50 & 1.50 \\ 1.50 & 1.50 & 1.50 \end{bmatrix}$	$\begin{bmatrix} 1.63 & 1.74 & 1.78 \\ 1.29 & 1.51 & 1.52 \end{bmatrix}$	$\begin{bmatrix} 0.10 & 0.10 & 0.09 \\ 0.12 & 0.09 & 0.13 \end{bmatrix}$
$\mathbf{\Phi}_1$	$\begin{bmatrix} 1.00 & 0.00 \\ 0.00 & 1.00 \end{bmatrix}$	$\begin{bmatrix} 1.00 & -0.01 \\ -0.01 & 1.40 \end{bmatrix}$	$\begin{bmatrix} 0.00 & 0.03 \\ 0.03 & 0.08 \end{bmatrix}$
$\mathbf{\Phi}_2$	$\begin{bmatrix} 1.00 & 0.00 \\ 0.00 & 0.70 \end{bmatrix}$	$\begin{bmatrix} 1.00 & 0.00 \\ 0.00 & 0.53 \end{bmatrix}$	$\begin{bmatrix} 0.00 & 0.05 \\ 0.05 & 0.08 \end{bmatrix}$
$\mathbf{\Omega}_1$	$\begin{bmatrix} 1.67 & 0.00 & 0.00 \\ 0.00 & 1.46 & 0.00 \\ 0.00 & 0.00 & 1.44 \end{bmatrix}$	$\begin{bmatrix} 1.66 & -0.01 & -0.01 \\ -0.01 & 1.46 & 0.01 \\ -0.01 & 0.01 & 1.43 \end{bmatrix}$	$\begin{bmatrix} 0.08 & 0.04 & 0.05 \\ 0.04 & 0.06 & 0.04 \\ 0.05 & 0.04 & 0.09 \end{bmatrix}$
$\mathbf{\Omega}_2$	$\begin{bmatrix} 0.75 & 0.00 & 0.00 \\ 0.00 & 0.82 & 0.00 \\ 0.00 & 0.00 & 0.90 \end{bmatrix}$	$\begin{bmatrix} 0.51 & -0.01 & -0.01 \\ -0.01 & 0.65 & 0.00 \\ -0.01 & 0.00 & 0.73 \end{bmatrix}$	$\begin{bmatrix} 0.09 & 0.04 & 0.04 \\ 0.04 & 0.08 & 0.06 \\ 0.04 & 0.06 & 0.07 \end{bmatrix}$

(c) Using hybrid approach (i.e., VGA approach followed by MCMC approach)

$\boldsymbol{\vartheta}_g$	True value	Means	Standard deviations
$\mathbf{M}_1$	$\begin{bmatrix} 6.20 & 6.20 & 6.20 \\ 6.20 & 6.20 & 6.20 \end{bmatrix}$	$\begin{bmatrix} 6.21 & 6.22 & 6.23 \\ 6.16 & 6.19 & 6.21 \end{bmatrix}$	$\begin{bmatrix} 0.05 & 0.06 & 0.04 \\ 0.06 & 0.09 & 0.04 \end{bmatrix}$
$\mathbf{M}_2$	$\begin{bmatrix} 1.50 & 1.50 & 1.50 \\ 1.50 & 1.50 & 1.50 \end{bmatrix}$	$\begin{bmatrix} 1.57 & 1.59 & 1.61 \\ 1.40 & 1.54 & 1.55 \end{bmatrix}$	$\begin{bmatrix} 0.06 & 0.06 & 0.06 \\ 0.09 & 0.06 & 0.08 \end{bmatrix}$
$\boldsymbol{\Phi}_1$	$\begin{bmatrix} 1.00 & 0.00 \\ 0.00 & 1.00 \end{bmatrix}$	$\begin{bmatrix} 1.00 & -0.01 \\ -0.01 & 1.40 \end{bmatrix}$	$\begin{bmatrix} 0.00 & 0.02 \\ 0.02 & 0.08 \end{bmatrix}$
$\boldsymbol{\Phi}_2$	$\begin{bmatrix} 1.00 & 0.00 \\ 0.00 & 0.70 \end{bmatrix}$	$\begin{bmatrix} 1.00 & 0.00 \\ 0.00 & 0.61 \end{bmatrix}$	$\begin{bmatrix} 0.00 & 0.05 \\ 0.05 & 0.08 \end{bmatrix}$
$\boldsymbol{\Omega}_1$	$\begin{bmatrix} 1.66 & 0.00 & 0.00 \\ 0.00 & 1.46 & 0.00 \\ 0.00 & 0.00 & 1.44 \end{bmatrix}$	$\begin{bmatrix} 1.67 & -0.01 & -0.01 \\ -0.01 & 1.47 & 0.01 \\ -0.01 & 0.01 & 1.44 \end{bmatrix}$	$\begin{bmatrix} 0.08 & 0.04 & 0.05 \\ 0.04 & 0.06 & 0.04 \\ 0.05 & 0.04 & 0.09 \end{bmatrix}$
$\boldsymbol{\Omega}_2$	$\begin{bmatrix} 0.75 & 0.00 & 0.00 \\ 0.00 & 0.82 & 0.00 \\ 0.00 & 0.00 & 0.90 \end{bmatrix}$	$\begin{bmatrix} 0.70 & -0.01 & -0.01 \\ -0.01 & 0.84 & 0.00 \\ -0.01 & 0.00 & 0.94 \end{bmatrix}$	$\begin{bmatrix} 0.08 & 0.06 & 0.05 \\ 0.06 & 0.07 & 0.08 \\ 0.05 & 0.08 & 0.08 \end{bmatrix}$

2 Performance on datasets generated from other mixture models

2.1 Simulation Setting 4 - Datasets generated from a mixture of independent Poisson distributions

Table 4: Means and variances of data generated from a mixture of independent Poisson distributions along with the average and standard deviations of the estimated parameters from the 25 datasets using VGA approach

$\boldsymbol{\vartheta}_g$	True value	Average Estimations	Standard deviations
Mean 1	$\begin{bmatrix} 1000 & 1500 & 1500 \\ 1000 & 1000 & 1000 \end{bmatrix}$	$\begin{bmatrix} 1000.09 & 1500.13 & 1499.86 \\ 999.65 & 1499.86 & 999.90 \end{bmatrix}$	$\begin{bmatrix} 1.29 & 1.57 & 1.64 \\ 1.19 & 1.94 & 1.23 \end{bmatrix}$
Mean 2	$\begin{bmatrix} 1000 & 1000 & 1000 \\ 1500 & 1000 & 1200 \end{bmatrix}$	$\begin{bmatrix} 999.83 & 999.67 & 999.41 \\ 1499.77 & 1000.04 & 1199.59 \end{bmatrix}$	$\begin{bmatrix} 1.18 & 1.49 & 1.44 \\ 1.42 & 1.16 & 1.03 \end{bmatrix}$
Variance 1	$\begin{bmatrix} 1000 & 1000 & 1000 \\ 1500 & 1000 & 1200 \end{bmatrix}$	$\begin{bmatrix} 999.84 & 999.67 & 999.41 \\ 1499.77 & 1000.04 & 1199.59 \end{bmatrix}$	$\begin{bmatrix} 1.18 & 1.49 & 1.44 \\ 1.42 & 1.16 & 1.03 \end{bmatrix}$
Variance 2	$\begin{bmatrix} 1000 & 1000 & 1000 \\ 1500 & 1000 & 1200 \end{bmatrix}$	$\begin{bmatrix} 999.84 & 999.67 & 999.41 \\ 1499.77 & 1000.04 & 1199.59 \end{bmatrix}$	$\begin{bmatrix} 1.18 & 1.49 & 1.44 \\ 1.42 & 1.16 & 1.03 \end{bmatrix}$

2.2 Simulation Setting 5 - Datasets generated from a mixture of independent negative binomial distributions.

Table 5: Means and variances of data generated from a mixture of independent negative binomial distributions along with the averages and standard deviations of the estimated parameters from the 25 datasets using VGA approach

$\boldsymbol{\vartheta}_g$	True value	Average Estimations	Standard deviations
Mean1	$\begin{bmatrix} 1000 & 500 & 1000 \\ 500 & 1000 & 500 \end{bmatrix}$	$\begin{bmatrix} 1000.23 & 500.57 & 999.79 \\ 500.05 & 999.79 & 500.28 \end{bmatrix}$	$\begin{bmatrix} 2.78 & 1.33 & 2.10 \\ 1.44 & 3.16 & 1.60 \end{bmatrix}$
Mean2	$\begin{bmatrix} 1000 & 1000 & 1000 \\ 500 & 500 & 1000 \end{bmatrix}$	$\begin{bmatrix} 999.08 & 999.66 & 999.05 \\ 500.56 & 500.01 & 1000.21 \end{bmatrix}$	$\begin{bmatrix} 4.91 & 5.58 & 4.96 \\ 3.19 & 2.47 & 4.80 \end{bmatrix}$
Variance1	$\begin{bmatrix} 11000 & 3000 & 11000 \\ 3000 & 11000 & 3000 \end{bmatrix}$	$\begin{bmatrix} 10253.70 & 2857.24 & 10294.979 \\ 2744.19 & 10118.551 & 2758.23 \end{bmatrix}$	$\begin{bmatrix} 320.34 & 92.38 & 361.92 \\ 85.82 & 288.34 & 87.19 \end{bmatrix}$
Variance2	$\begin{bmatrix} 11000 & 11000 & 11000 \\ 3000 & 3000 & 11000 \end{bmatrix}$	$\begin{bmatrix} 10406.78 & 10414.11 & 10732.97 \\ 2758.45 & 2751.98 & 10321.68 \end{bmatrix}$	$\begin{bmatrix} 674.86 & 540.25 & 584.69 \\ 172.91 & 139.36 & 480.22 \end{bmatrix}$

2.3 Simulation Setting 6 - Datasets generated from an $G = 8$ component MVPLN distributions

Table 6: The Frobenius norm of the difference of the estimated parameters and true value of the parameters.

(a) Using VGA approach

	$g=1$	$g=2$	$g=3$	$g=4$	$g=5$	$g=6$	$g=7$	$g=8$
$\|\|\mathbf{M}_g - \widehat{\mathbf{M}}_g\|\|_F$	0.71	0.68	0.55	0.62	0.61	0.58	0.63	1.31
$\|\|\boldsymbol{\Phi}_g - \widehat{\boldsymbol{\Phi}}_g\|\|_F$	1.47	0.82	0.93	0.94	1.59	1.61	0.86	9.70
$\|\|\boldsymbol{\Omega}_g - \widehat{\boldsymbol{\Omega}}_g\|\|_F$	0.55	0.64	0.34	0.58	0.80	0.36	0.58	1.19

(b) Using Hybrid Approach

	$g=1$	$g=2$	$g=3$	$g=4$	$g=5$	$g=6$	$g=7$	$g=8$
$\|\|\mathbf{M}_g - \widehat{\mathbf{M}}_g\|\|_F$	1.44	1.60	1.69	1.69	1.58	1.59	1.48	2.01
$\|\|\boldsymbol{\Phi}_g - \widehat{\boldsymbol{\Phi}}_g\|\|_F$	2.91	3.90	1.73	3.89	4.48	4.81	5.06	3.92
$\|\|\boldsymbol{\Omega}_g - \widehat{\boldsymbol{\Omega}}_g\|\|_F$	2.26	3.43	1.16	1.71	9.74	8.19	6.00	1.22

Note: MCMC based approach was not used here due to computational cost with the MCMC based approach on a $G = 8$ component model (see Section 3 for more details on computational time of MCMC based approach.

3 Computational Times

The average computational time along with the standard deviation to fit the mixtures of MVPLN models using all three approaches (MCMC based approach, VGA based approach, and hybrid approach) are summarized below. The average and standard deviations are computed using the 25 datasets under each of the Simulation 1, 2, and 3 where the datasets were generated from the mixtures of MVPLN model. We do not provide the computational time for Simulation 4 and 5 as the datasets were generated using other models. Due to the computational cost of fitting MCMC based approach, we only provide comparison of times for fitting the model with the correct number of components.

Table 7: Means and standard deviations of the computational times (in minutes) for fitting the mixtures of MVPLN with the correct number of components on all 25 datasets from Simulation 1, 2, and 3.

	**Approach**	**Average time (in minutes)**	**Standard Deviation (in minutes)**
Simulation 1	MCMC	608.0079	53.5589
	VGA	0.2337	0.0731
	Hybrid	8.7897	0.0033
Simulation 2	MCMC	2504.1690	260.9368
	VGA	2.1000	0.2355
	Hybrid	9.6099	0.0048
Simulation 3	MCMC	2736.1360	218.3634
	VGA	0.3486	0.1075
	Hybrid	5.5503	0.0031

4 The $\boldsymbol{\Sigma}_g = \boldsymbol{\Phi}_g \otimes \boldsymbol{\Omega}_g$ for Simulation 1, 2 and 3.

Simulation 1

$$\boldsymbol{\Sigma} = \begin{bmatrix} 1.66 & -0.61 & 0.77 & -0.91 & 0.34 & -0.42 \\ -0.61 & 1.46 & 0.17 & 0.34 & -0.80 & -0.09 \\ 0.77 & 0.17 & 1.44 & -0.42 & -0.09 & -0.79 \\ -0.91 & 0.34 & -0.42 & 2.11 & -0.78 & 0.98 \\ 0.34 & -0.80 & -0.09 & -0.78 & 1.86 & 0.22 \\ -0.42 & -0.09 & -0.79 & 0.98 & 0.22 & 1.83 \end{bmatrix}$$

Simulation 2

$$\boldsymbol{\Sigma}_1 = \begin{bmatrix} 1.66 & -1.04 & -0.61 & 0.38 & 0.77 & -0.48 \\ -1.04 & 2.33 & 0.38 & -0.86 & -0.48 & 1.08 \\ -0.61 & 0.38 & 1.46 & -0.91 & 0.17 & -0.11 \\ 0.38 & -0.86 & -0.91 & 2.05 & -0.11 & 0.24 \\ 0.77 & -0.48 & 0.17 & -0.11 & 1.44 & -0.90 \\ -0.48 & 1.08 & -0.11 & 0.24 & -0.90 & 2.02 \end{bmatrix}$$

$$\boldsymbol{\Sigma}_2 = \begin{bmatrix} 1.00 & 0.00 & 0.00 & 0.00 & 0.00 & 0.00 \\ 0.00 & 1.08 & 0.00 & 0.00 & 0.00 & 0.00 \\ 0.00 & 0.00 & 1.04 & 0.00 & 0.00 & 0.00 \\ 0.00 & 0.00 & 0.00 & 1.13 & 0.00 & 0.00 \\ 0.00 & 0.00 & 0.00 & 0.00 & 1.10 & 0.00 \\ 0.00 & 0.00 & 0.00 & 0.00 & 0.00 & 1.19 \end{bmatrix}$$

Simulation 3

$$\boldsymbol{\Sigma}_1 = \begin{bmatrix} 1.66 & 0.00 & 0.00 & 0.00 & 0.00 & 0.00 \\ 0.00 & 2.33 & 0.00 & 0.00 & 0.00 & 0.00 \\ 0.00 & 0.00 & 1.46 & 0.00 & 0.00 & 0.00 \\ 0.00 & 0.00 & 0.00 & 2.05 & 0.00 & 0.00 \\ 0.00 & 0.00 & 0.00 & 0.00 & 1.44 & 0.00 \\ 0.00 & 0.00 & 0.00 & 0.00 & 0.00 & 2.02 \end{bmatrix}$$

$$\mathbf{\Sigma}_2 = \begin{bmatrix} 0.75 & 0.00 & 0.00 & 0.00 & 0.00 & 0.00 \\ 0.00 & 0.52 & 0.00 & 0.00 & 0.00 & 0.00 \\ 0.00 & 0.00 & 0.82 & 0.00 & 0.00 & 0.00 \\ 0.00 & 0.00 & 0.00 & 0.57 & 0.00 & 0.00 \\ 0.00 & 0.00 & 0.00 & 0.00 & 0.90 & 0.00 \\ 0.00 & 0.00 & 0.00 & 0.00 & 0.00 & 0.63 \end{bmatrix}$$
