# Supplementary material for: Finite mixtures of matrix variate Poisson-log normal distributions for three-way count data
Source: Bioinformatics. 2023 Apr 5;39(5):btad167. doi: 10.1093/bioinformatics/btad167 (PMC10159656; doi:10.1093/bioinformatics/btad167)
Supplement: btad167_Supplementary_Data [file btad167_supplementary_data.zip › Supplementary File/Supplementary_File_3.pdf]

1 MCMC based approach

1.1 Convergence

To determine whether the MCMC chains have converged to the posterior distribution, two diagnostic criteria are used. One is the *potential scale reduction factor* (Gelman and Rubin, 1992) and the other is the *effective number of samples* (Gelman *et al.*, 2013). The algorithm for mixtures of MVPLN distributions is set to check if the `RStan`-generated chains have a *potential scale reduction factor* less than 1.1 and an *effective number of samples* value greater than 100 (Annis *et al.*, 2016). If both criteria are met, the algorithm proceeds. Otherwise, the chain length is set to increase by 100 iterations and sampling is redone. The algorithm is set to run a minimum of three chains at once, as recommended by Annis *et al.* (2016). However, the user is able to specify more chains within the algorithm. According to Neath (2012), the Monte Carlo sample size should be increased with the MCMC-EM iteration count due to persistent Monte Carlo error, which can contribute to slow or no convergence. For the algorithm for mixtures of MVPLN distributions, the number of `RStan` iterations is set to start with a modest number of 1000 and is increased with each MCMC-EM iteration as the algorithm proceeds. To check whether the likelihood has reached its maximum, the Heidelberger and Welch's convergence diagnostic (Heidelberger and Welch, 1983) is applied to all log-likelihood values after each MCMC-EM iteration, using a significance level of 0.05. This diagnostic is implemented via the `heidel.diag` function in `coda` package (Plummer *et al.*, 2006). If not converged, further MCMC-EM iterations are performed until convergence is reached.

1.2 Initialization

For initialization of the $z_{ng}^{(t)}$, two approaches are used: k-means and random initialization. For k-means initialization, k-means clustering is performed on the dataset and the resulting cluster memberships are used for the initialization of $z_{ng}^{(t)}$. For random initialization, random values are chosen for $z_{ng}^{(t)} \in [0, 1]$ such that $\sum_{g=1}^{G} z_{ng} = 1$ for all n. If multiple initialization runs are considered, the $z_{ng}^{(t)}$ values corresponding to the run with the highest log-likelihood values are used for downstream analysis. For initialization of $\mathbf{M}_g$, the logarithm of the cluster-specific mean is used. Then $\mathbf{\Phi}_g$ and $\mathbf{\Omega}_g$ are initialized as $\mathbf{\Phi}_g = \mathbf{I}_{r\times r}$ and $\mathbf{\Omega}_g = \mathbf{I}_{p\times p}$, respectively.

1.3 Parallel implementation

Coarse grain parallelization has been deployed in the context of model-based clustering via Gaussian mixtures (McNicholas *et al.*, 2010). When a range of clusters are considered for a dataset, i.e., $G_{\min}$:$G_{\max}$, each G is independent and there is no dependency between them. Therefore, the algorithms corresponding to $G_{\min}$:$G_{\max}$ can be run in parallel, each one on a different processor. Here, the algorithm for the mixtures of MVPLN distributions is parallelized using the `parallel` package (R Core Team, 2017) and the `foreach` package (Revolution Analytics and Weston, 2015). All analyses were done using the parallelized code.

2 VGA based approach

2.1 Convergence

To determine convergence, we utilized a criterion based on the Aitken acceleration criterion (Aitken, 1926). The model is assumed to be converged at iteration m if $l_\infty^{(m+1)} - l_\infty^{(m)}$ is positive and smaller than some ϵ, where $l^{(m)}$ is the value of the log-likelihood at iteration m and $l_\infty^{(m+1)}$ is an asymptotic estimate, at iteration $m+1$, of the log-likelihood given by

$$l_\infty^{(m+1)} = l^{(m)} + \frac{l^{(m+1)} - l^{(m-1)}}{1 - a^{(m)}},$$

where

$$a^{(m)} = \frac{l^{(m+1)} - l^{(m)}}{l^{(m)} - l^{(m-1)}}$$

(Böhning *et al.*, 1994). Here, we used $\epsilon = 0.05$.

2.2 Initialization

For initialization of the $\hat{Z}_{ng}^{(t)}$, we used k-means initialization. k-means clustering is performed on the dataset with 100 different initial partitioning and the resulting cluster memberships are used for the initialization of $\hat{Z}_{ng}^{(t)}$. For initialization of $\mathbf{M}_g$, the logarithm of the cluster-specific mean is used. Then $\mathbf{\Phi}_g$ and $\mathbf{\Omega}_g$ are initialized as $\mathbf{\Phi}_g = \mathbf{I}_{r\times r} \times \sqrt{\min \operatorname{diag}\{\operatorname{Var}(\textbf{vec\_logY})\}}$ and

$\mathbf{\Omega}_g = \mathbf{I}_{p\times p} \times \sqrt{\min \text{diag}(\text{Var}(\textbf{vec\_logY}))}$, respectively where **vec_logY** is the $rp \times N$-dimensional matrix obtained by using the vectorized log-transformed $\mathbf{Y}_n$ as the nth row and the diag$\{\cdot\}$ puts the diagonal elements of the $rp \times rp$ matrix into a rp-dimensional vector. Variational parameter $\boldsymbol{\xi}_{ng}$ is initialized as the log-transformed $\mathbf{Y}$ and $\mathbf{\Delta}_g$ and $\boldsymbol{\kappa}_g$ are initialized as $\mathbf{\Delta}_g = \mathbf{I}_{r\times r} \times 0.0001$ and $\mathbf{\Omega}_g = \mathbf{I}_{p\times p} \times 0.0001$, respectively.

References

Aitken, A. C. (1926). A series formula for the roots of algebraic and transcendental equations. *Proceedings of the Royal Society of Edinburgh*, **45**, 14–22.

Annis, J., Miller, B. J., and Palmeri, T. J. (2016). Bayesian inference with Stan: A tutorial on adding custom distributions. *Behavior Research Methods*, **49**, 1–24.

Böhning, D., Dietz, E., Schaub, R., Schlattmann, P., and Lindsay, B. (1994). The distribution of the likelihood ratio for mixtures of densities from the one-parameter exponential family. *Annals of the Institute of Statistical Mathematics*, **46**, 373–388.

Gelman, A. and Rubin, D. B. (1992). Inference from iterative simulation using multiple sequences. *Statistical Science*, **7**, 457–472.

Gelman, A., Carlin, J. B., Stern, H. S., Dunson, D. B., Vehtari, A., and Rubin, D. B. (2013). *Bayesian Data Analysis*. Chapman & Hall/CRC Press, Boca Raton, FL.

Heidelberger, P. and Welch, P. D. (1983). Simulation run length control in the presence of an initial transient. *Operations Research*, **31**, 1109–1144.

McNicholas, P. D., Murphy, T. B., McDaid, A. F., and Frost, D. (2010). Serial and parallel implementations of model-based clustering via parsimonious Gaussian mixture models. *Computational Statistics and Data Analysis*, **54**, 711–723.

Neath, R. C. (2012). On convergence properties of the Monte Carlo EM algorithm. arXiv preprint arXiv:1206.4768.

Plummer, M., Best, N., Cowles, K., and Vines, K. (2006). CODA: Convergence diagnosis and output analysis for MCMC. *R News*, **6**, 7–11. R package version 0.19-1.

R Core Team (2017). *R: A Language and Environment for Statistical Computing*. R Foundation for Statistical Computing, Vienna, Austria.

Revolution Analytics and Weston, S. (2015). *foreach: Provides Foreach Looping Construct for R*. R package version 1.4.3.
